# Carbon Dots in Bioimaging, Biosensing and Therapeutics: A Comprehensive Review

**DOI:** 10.1002/smsc.202200012

**Published:** 2022-05-08

**Authors:** Boyang Wang, Huijuan Cai, Geoffrey I. N. Waterhouse, Xiaoli Qu, Bai Yang, Siyu Lu

**Affiliations:** ^1^ Green Catalysis Center College of Chemistry Zhengzhou University Zhengzhou 450000 China; ^2^ School of Chemical Sciences The University of Auckland Auckland 1142 New Zealand; ^3^ Erythrocyte Biology Laboratory School of Life Sciences Zhengzhou University Zhengzhou 450001 China; ^4^ State Key Lab of Supramolecular Structure and Materials College of Chemistry Jilin University Changchun 130012 China

**Keywords:** bioapplications of carbon dots, photothermal therapy, photodynamic therapy, properties of carbon dots, synthesis of carbon dots

## Abstract

Carbon dots (CDs), comprising crystalline graphitized carbon cores and polymer surface groups, are currently attracting a lot of interest in biological fields owing to their fluorescent properties, high photostability, biocompatibility and low toxicity. In addition, the easy preparation and functionalization of CDs stimulate the development of CDs‐based composite materials with specific functions. Presently, the biological applications of CDs are growing at a remarkable speed, justifying the need for up‐to‐date review articles that capture recent progress in this blossoming field. In this review, breakthroughs in the synthesis, modification, optical properties, toxicology and biocatalytic platforms of CDs are described. Further, recent research related to bioimaging, biosensing, drug delivery, antibacterial, anticancer (photothermal therapy, photodynamic therapy and synergistic therapy) and antiviral therapies involving CDs are discussed in detail. Finally, a perspective on the prospects and challenges of CDs in the fields of biomedicine and biotechnology is provided.

## Graphical abstract

The development and application of new functional nanomaterials have always been the focus of various research fields. Among them, carbon dots (CDs) are the newest member of the carbon‐based nanomaterials, which has aroused great interest in bioapplications due to its extraordinary optical and biological properties. Therefore, it is important to make a comprehensive summary of its wide bioapplication values.

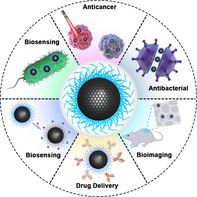

## 1 Introduction

Carbon dots (CDs) are a vibrant collection of fluorescent carbon nanomaterials, possessing a small crystalline graphitized core and polymer surface groups.^[^ ^1^, ^2^, ^3^ ^]^ Due to their structure, CDs are endowed with unique optical and electronic properties^[^ ^4^, ^5^, ^6^ ^]^ including a very high fluorescence quantum yield, red/near infrared emission, excellent stability and dispersion properties.^[^ ^7^, ^8^, ^9^ ^]^ Not surprisingly, CDs are finding increasing utilization in the fabrication of electronics, energy conversion devices and sensors.^[^ ^10^, ^11^, ^12^ ^]^ In addition, due to their unique chemical, physical and optical properties, CDs and CDs‐based nanomaterials hold the greatest promise for implementation in a wide array of biological and biomedical applications, especially imaging and therapeutics.

The past decade has seen a proliferation in the biological and biomedical applications using CDs, owing to small size (1–10 nm), unique optical properties, easy functionalization, and biocompatibility.^[^ ^13^, ^14^, ^15^ ^]^ The small size allows them to traverse various natural biological barriers in vivo including ion channels, blood‐brain barrier (BBB) and the glomerular filtration barrier.^[^ ^16^, ^17^, ^18^ ^]^ Their tunable functional properties make them ideal nanocapsules and nanocarriers to load and deliver drugs and genes to specific targets in vivo.^[^ ^19^, ^20^ ^]^ Moreover, the optical properties of some CDs creates opportunities in biomedical applications.^[^ ^21^, ^22^, ^23^ ^]^ For example, many CDs absorb strongly in near‐infrared region, delivering in‐situ photothermal effects for photoacoustic imaging and photothermal therapy.^[^ ^24^, ^25^, ^26^ ^]^ CDs are also being used as fluorescent contrast agents for deep‐tissue fluorescence imaging.^[^ ^27^, ^28^ ^]^

To date, the have been many excellent reviews on the synthesis, chemical and physical properties and their biological applications of CDs. However, most focus on a single aspect (synthesis, properties or biological application), rather than providing a comprehensive overview over all these areas. Further, the general field of CDs is evolving rapidly, especially in the biological and biomedical areas. About 40% of all papers published on CDs over the past 5 years relate to bioapplications of CDs or their composite materials. Regular summaries of recent progress in the application of CDs in biological/biomedical fields is therefore essential for mapping the future directions of this exciting field, necessitating regular comprehensive review articles.

In the current review, a comprehensive and heuristic summary of CDs is provided, covering the synthesis of CDs and CDs‐based materials, their unique chemical and physical properties, and their recent applications in biological/biomedical fields. Specifically, we will focus on the use of CDs in bioimaging, biosensing, drug delivery, antibacterial applications, anticancer therapies (photothermal therapy, photodynamic therapy and synergistic therapy) and antiviral treatments. In addition, we will discuss current trends, future directions, and persisting controversies in this field. We hope that this review will stimulate further interest in CDs and CDs‐based composites, by showcasing their numerous advantages in a broad spectrum of bio‐related applications.

## 2 Structure of CDs

The structure of CDs determines their application potential. A large number of studies have shown that CDs are core‐shell structure, usually without a clear boundary between the carbon core and the polymer shell.^[^ ^29^, ^30^, ^31^ ^]^ The core can often contain polycrystalline nanodomains containing tiny carbon clusters surrounded by amorphous domains. Carbon clusters as subdomains in the carbon core can have a conjugated π‐structure or a diamond‐like structure, which can be judged using the lattice observed in the transmission electron microscope (TEM). The presence of the polymer surface endows the CDs with specific properties. The polymer side chains can usually be detected utilizing atomic force microscope (AFM) and dynamic light scattering (DLS).^[^ ^32^ ^]^ The presence of the side chains is confirmed by the comparison of particle sizes. The existence of the core‐shell structure was further confirmed by comparing the structural parameters of the CDs with the model‐fitted corresponding small‐angle X‐Ray scattering (SAXS) modes recently.^[^ ^33^ ^]^

### 2.1 Morphology and Size of CDs

Although most CDs exhibit dot‐like structures, researchers have developed CDs with different sizes and morphologies (triangles, ribbons, rods, etc.) through precursor selection and reaction process design.^[^ ^34^, ^35^, ^36^, ^37^ ^]^ The symmetrical precursor phloroglucinol was used as the precursor, and an appropriate amount of concentrated sulfuric acid was added to the ethanol solution as a catalyst to control the size of CDs. Due to the presence of three highly active hydrogen atoms in the three meta positions activated by the three electron‐donating hydroxyl groups in a single molecule, the as‐prepared CDs exhibit a unique triangular structure. The emission wavelength is red‐shifted with increasing size, as expected from quantum confinement effects.^[^ ^38^ ^]^ The same group used *p*‐phenyldiacetonitrile with a cyano group as a precursor and formed carbon quantum rings of different diameters by linking curved carbon quantum ribbons of different lengths. The results show that cyano groups are inducing carbon quantum band bending. Interestingly, the as‐prepared CDs also exhibit size dependence, with the bandgap gradually decreasing with increasing size.^[^ ^39^ ^]^

### 2.2 Surface Functional Groups of CDs

During the preparation process of CDs, various functional groups such as –OH, –COOH, –CHO, –NH_2_ and –SH can be introduced on the surface of CDs according to the different kinds of precursors.^[^ ^40^, ^41^, ^42^ ^]^ The type and number of functional groups can affect their properties. Usually, oxygen‐containing functional groups will make CDs negatively charged, while N‐containing functional groups will make CDs positively charged, and the difference in charge will endow CDs with different passivation capabilities. Therefore, it is necessary to know the relative number of functional groups on the surface of CDs. The determination of the number of amino groups is usually performed by ninhydrin colorimetry, selecting lysine as the standard solution to obtain a standard curve, and using an ultraviolet spectrometer to measure the spectrum of the resulting solution at 560 nm. The carboxyl group content can be determined by Boehm titration by mixing CDs and NaHCO_3_ solution, and then the mixed solution is titrated with HCl, with pH as the indicated end point.^[^ ^43^ ^]^ In another experiment, Kang et al. performed alkali titration according to standard methods to measure the total amount of hydroxyl and carboxylic acid, and then conducted conductometric titration to understand the relative content of hydroxyl and carboxylic acid, and finally calculated their respective contents.^[^ ^44^ ^]^

## 3 Preparation of CDs

To date, a wide range of synthetic routes have been developed for the preparation of CDs (**Table** **1**). Most syntheses seek simple, cost‐effective, size‐controlled or scalable methods towards CDs of high quality. The synthetic approaches are generally classified into two main categories: top‐down and bottom‐up.^[^ ^45^, ^46^, ^47^ ^]^

**Table 1:** Different “top‐down” and “bottom‐up” approaches in synthesizing CDs

Methods	Carbon source	Advantage	Disadvantage	Discovery time
Laser ablation	SWCNTs	Easy to manipulate the size of CDs	High cost and sophisticated process	2004^[^ ^50^ ^]^
Arc discharge	graphite powder and cement	Large scale preparation	Low quantum yield and low purity	2006^[^ ^293^ ^]^
Chemical oxidation	Candle soot	High yield, high purity	Environmental pollution and low quantum yield	2007^[^ ^294^ ^]^
Electrochemical oxidation	MWCNTs	Easy to manipulate the size of CDs	Required longer time	2007^[^ ^295^ ^]^
Pyrolysis	Citrate	Easy operation, less time consuming	Broad size distribution	2008^[^ ^296^ ^]^
Template	F127	Easy to manipulate the size of CDs	Cumbersome steps	2009^[^ ^71^ ^]^
Microwave	PEG‐200 and saccharide	Rapid and constant volumetric heating	High energy cost, uneven heating	2009^[^ ^297^ ^]^
Hydrothermal/Solvothermal	L‐Ascorbic acid	Cheap, eco‐friendly, controllable	Low yield, low purity	2010^[^ ^298^ ^]^
Organic approach	p‐bromobenzoic acid	Easy to manipulate the size and functional group of CDs	Complex process, harsh reaction conditions	2010^[^ ^77^ ^]^
Sonochemistry/ultrasonication	Glucose	Mild experimental conditions, green energy sources	Low yield, not easy to dope	2011^[^ ^79^ ^]^

### 3.1 Top down

**Figure 1:**
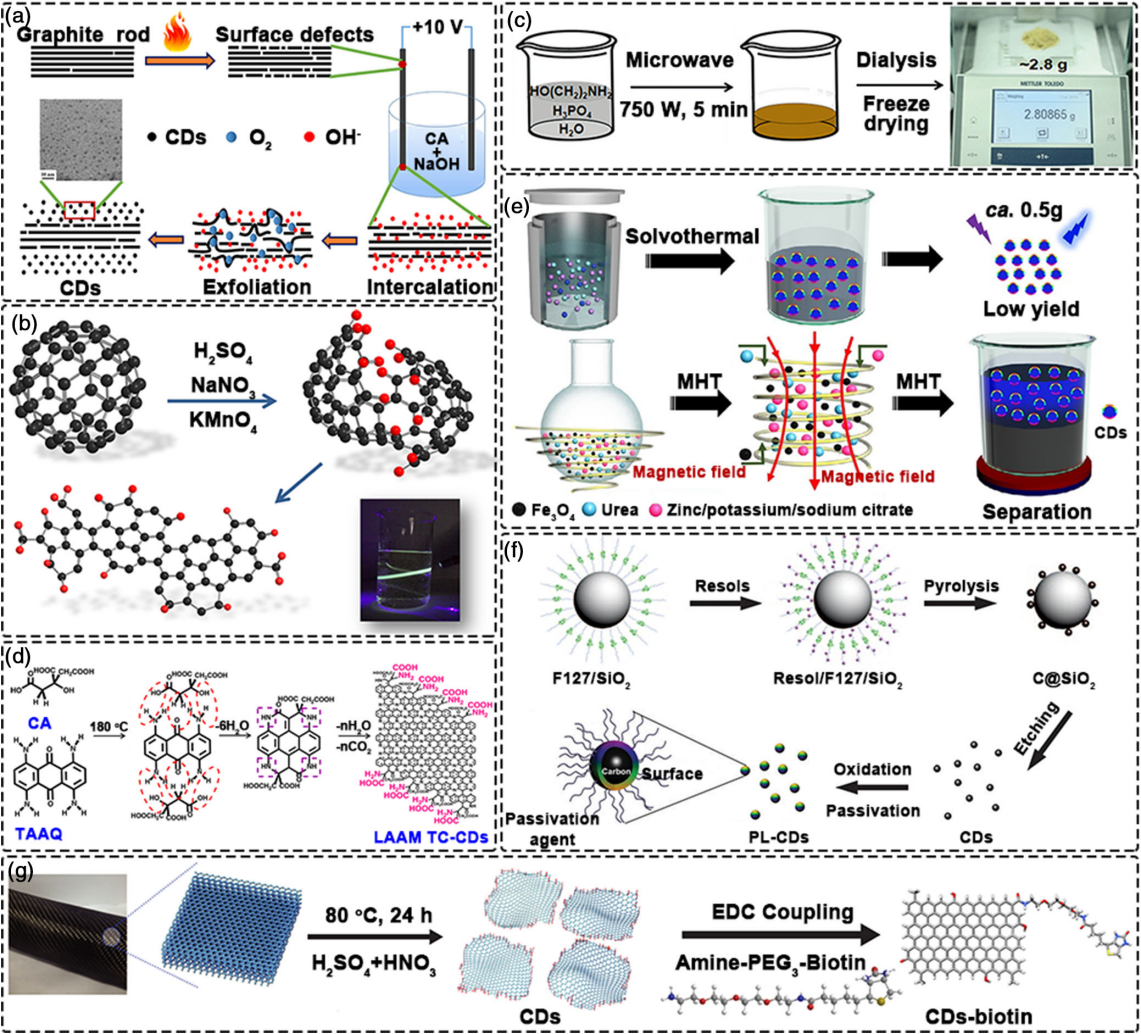
Synthesis of CDs by top‐down methods, a) Electrochemical oxidation. Reproduced with permission.^[^ ^52^ ^]^ Copyright 2017, American Chemical Society. b) Acid reflux method. Reproduced with permission.^[^ ^54^ ^]^ Copyright 2015, American Chemical Society. Synthesis of CDs by bottom‐up methods, c) Microwave method. Reproduced with permission.^[^ ^57^ ^]^ Copyright 2018, Wiley‐VCH. d) Hydrothermal method. Reproduced with permission.^[^ ^64^ ^]^ Copyright 2020, Springer Nature. e) Large‐scale preparation of nanomedicine used CDs. Reproduced with permission.^[^ ^65^ ^]^ Copyright 2020, Wiley‐VCH. f) Template method. Reproduced with permission.^[^ ^71^ ^]^ Copyright 2009, Wiley‐VCH. g) Surface modification of CDs by Biotin. Reproduced with permission.^[^ ^95^ ^]^ Copyright 2018, Springer Nature.

Top‐down methods are based on the fragmentation of larger carbon structures, encompassing arc discharge, laser ablation, oxidative cracking, and electrochemical oxidation.^[^ ^48^, ^49^ ^]^ In 2004, Xu et al. isolated an unknown fluorescent carbon material when purifying single‐walled carbon nanotubes made from arc‐discharge soot. This is considered the first discovery of CDs.^[^ ^50^ ^]^ The disadvantage of this method is that the CDs size is not uniform, with separation/purification difficult due to the co‐existence of a mixture of differently sized carbon nanostructures. Further, the CDs yield by this approach is extremely low. Laser ablation and oxidative cracking use strong acid passivation of the surface of CDs. Owing to the toxic reagents required, there have been surpassed by electrochemical routes. The electrochemical oxidation method typically uses electrolytic graphite rods as a carbon source and was first proposed by Kang et al.^[^ ^51^ ^]^ The obtained CDs possess a highly crystalline nature and offer high photocatalytic activity under visible‐light irradiation. Using this strategy, the properties of the prepared CDs can be regulated simply by changing the electrolyte (**Figure** **1**a).^[^ ^52^ ^]^ Trajkovic et al. observed that CDs prepared by the electrochemical route could reduce immune‐mediated fulminant hepatitis by decreasing liver inflammation, oxidative stress, apoptosis and autophagy.^[^ ^53^ ^]^ CDs exerted an immune regulation effect by interfering with the activation of T cells and macrophages, as well as a direct hepatoprotective effect due to liver accumulation. However, the CDs prepared by electrolysis have few surface functional groups, resulting in generally poor water dispersibility. Therefore, for biological applications, electrochemically‐synthesized CDs typically need surface functionalization via reflux methods in strongly acidic media. Typically, concentrated HNO_3_ or a mixture of concentrated HNO_3_ and H_2_SO_4_ are used owing to their ability to oxidize graphite, C_60_ and carbon rods, thus imparting CDs with excellent water dispersibility (Figure 1b).^[^ ^54^ ^]^ For example, the CDs modified by the reflux method were successfully used to construct a fluorescent sensing platform after tyramine functionalization. This platform was able to detect a series of metabolites with high sensitivity and specificity.^[^ ^55^ ^]^ The developed CDs also possessed a catalytic activity as peroxidase mimics. The main advantage of top‐down approaches for the preparation of CDs is that abundant the raw materials (i.e., graphite) are abundant, thus allowing the large‐scale production of CDs of a highly crystalline nature (especially if electrochemical routes are used). However, top‐down methods generally suffer from a nonuniform CDs morphology, broad size distributions, and may contain impurities which can quench fluorescence.

### 3.2 Bottom up

Bottom‐up methods involve the construction of CDs from simple carbon precursors including organic molecules or polymers. Through dehydration reactions and further carbonization processes, CDs are produced. The precursors generally possess –OH, –COOH, and ‐NH_2_ groups, favoring the formation of CDs via dehydration and carbonization processes at elevated temperatures. Owing to their simple operation and well‐defined carbon precursors, bottom‐up techniques are now widely employed to produce CDs with uniform morphologies, narrow size distributions and stable properties. Common bottom‐up methods include microwave syntheses and solvothermal/hydrothermal processes. In addition, template‐assisted, cage opening or various other methods have also been reported for the bottom‐up preparation of CDs, as discussed below.

#### 3.2.1 Microwave

Microwave treatment causes the dehydration and pyrolysis of reaction precursors, eventually leading to carbonization into CDs.^[^ ^56^ ^]^ Microwave methods have the advantage of very short reaction times. Initially, microwave reactors were used. However, subsequent studies found that microwave ovens can also be used to prepare CDs. The CDs synthesized by microwave techniques are usually uniform in size, with the size being closely related to with microwave power and treatment time. Due to its fast‐heating rate and accelerated reaction kinetics, microwave technologies offer an efficient route for CDs syntheses. Further, the combination of microwave heating and traditional processing methods such as pyrolysis, hydrothermal/solvothermal and chemical oxidation can effectively shorten the reaction time. As an example, Lin et al. used the microwave method to produce gram‐quantities of CDs in high‐yield within a several minutes (Figure 1c).^[^ ^57^ ^]^ Prato et al. used arginine and ethylenediamine as raw materials for preparing CDs via the microwave method.^[^ ^58^ ^]^ The effects of different parameters (such as microwave power, time, etc.) and common post‐processing steps to purify CDs were discussed in detail. Using the microwave method, Rigodanza et al. systematically explored the changes in the structure, chemical and photophysical characteristics of the CDs during the synthesis process, and determined that the formation of CDs involved four consecutive steps: 1) aggregation of small organic molecules; 2) formation of a dense core with an expanded shell; 3) collapse of the shell; and 4) aromatization of the core.^[^ ^33^ ^]^

#### 3.2.2 Hydrothermal/Solvothermal

Whilst microwave methods are advantageous due to their short synthesis times, such rapid reactions can produce a lot of unwanted by‐products, thereby requiring extensive CDs purification. Accordingly, hydrothermal/solvothermal methods have surpassed microwave methods in popularity, and are now the most common method for preparing CDs. In addition, the surfaces of the CDs prepared by this method are usually rich in hydrophilic functional groups (hydroxyl, carboxyl, amino, etc.), which greatly enhances the water dispersibility of the CDs and expands the scope of application of CDs in biological fields.^[^ ^59^, ^60^, ^61^ ^]^ Current applications of hydrothermally/solvothermally‐synthesized CDs includes imaging, sensing, and therapeutics.^[^ ^62^, ^63^ ^]^ Using the hydrothermal method and citric acid and 1,4,5,8‐tetraminoanthraquinone as carbon sources, Fan et al. successfully prepared CDs rich in α‐carboxyl and amino groups (Figure 1d).^[^ ^64^ ^]^ CDs mimic large amino acids in structure and can be loaded with aromatic drugs through π‐π stacking interactions, thereby allowing the targeted delivery of chemotherapeutic drugs to tumors. Using a magnetic‐assisted solvothermal method, Chen et al. synthesized CDs on a large‐scale (up to 85 g) in 1 h (Figure 1e).^[^ ^65^ ^]^ The CDs obtained could attach to polymer chains through hydrogen bonding and electrostatic interactions to form a nanofiber scaffold, thereby improving wound healing efficiencies.

#### 3.2.3 Template‐Assisted

Considering that multiple‐step purification and size‐enrichment processes are frequently required to obtain CDs of a desired size and size‐distribution,^[^ ^66^, ^67^ ^]^ template‐assisted syntheses have been developed that endow CDs with specific morphologies and a uniform size distribution. Templates used vary from soft matter (e.g., surfactants)^[^ ^68^, ^69^ ^]^ to hard materials such as silica,^[^ ^70^, ^71^ ^]^ zeolites,^[^ ^72^ ^]^ metal‐organic framework,^[^ ^73^ ^]^ and layered host matrices.^[^ ^74^ ^]^ For hard template approaches, CDs are usually formed in a restrained/confined space and acid or alkali treatments then used to selectively etch away the template to release the CDs. Further, a combination of soft (e.g., Pluronic P123) and hard template (e.g., ordered MSNs SBA‐15) strategies can be used to prepare CDs with specific sizes, compositions and crystalline degrees, thus allowing the creation of CDs with precisely tailored properties.^[^ ^75^ ^]^

#### 3.2.4 Other Methods

Although some common methods for preparing CDs have been mentioned in this section, CDs can also be synthesized by another bottom‐up synthesis. In the early stage, researchers adopted organic synthesis methods for the controllable preparation of CDs. This method allows precise control over the chemical structure and size of CDs.^[^ ^76^, ^77^, ^78^ ^]^ However, this preparation method involves a complicated multi‐step procedure, uses environmentally unfriendly organic solvents/ligands/acids under extremely harsh reaction conditions, and the quantum yield (QYs) of the obtained CDs are generally poor, so it is gradually eliminated. The sonication process creates alternating pressure differentials in the solvent, promoting the formation of transient vacuum microbubbles.^[^ ^79^ ^]^ Subsequently, these microbubbles grow by rectifying diffusion and coalescence to reach a critical size. After reaching the maximum size, these bubbles burst violently with localized temperatures as high as 2000–5000 K, pressures as high as 20 MPa, thereby promoting precursor polymerization and carbonization.^[^ ^80^ ^]^ In addition to organic solvents, changing parameters such as ultrasonic time, amplitude and temperature can also achieve the preparation of CDs of different sizes by ultrasonic PEG‐400.^[^ ^81^ ^]^

### 3.3 Surface Modification/Doping

An important consideration in engineering CDs with desired functions bioapplications is their composition (including surface functional groups). Various synthetic and post‐synthetic strategies are used for this purpose.^[^ ^82^ ^]^ Advances in synthesis and surface modification technologies make CDs appealing platforms for engineering of biological probes with improved brightness, tunable fluorescence, enhanced water‐solubility, and biocompatibility.^[^ ^83^, ^84^, ^85^ ^]^ For example, heteroatom doping can endow CDs regulated structural defects and enhanced optical properties.^[^ ^86^, ^87^ ^]^ These new surface states inhibit or eliminate the original *O*‐states and promote radiative recombination, leading to higher QYs and doped CDs with excitation‐independent emissions.^[^ ^84^ ^]^ In addition to intrinsic property control achieved by element doping, post‐synthetic modifications involving non‐covalent and covalent coupling strategies can be used to further tune the functional properties of CDs (**Table** **2**).^[^ ^88^, ^89^ ^]^

**Table 2:** Different passivation methods of CDs

Precursors	Passivated form	New Performance	Advantage	Disadvantage	Application	Ref.
Citric acid TAAQ	N doping	Mimic large amino acids	Abundant raw materials, simple preparation, and high QYs	Uncontrollable, single function	Bioimaging and Drug Delivery	[64]
Polythiophene phenylpropionic acid	S doping	Broad absorption			PA imaging and PTT	[265]
Phenylboronic acid	B doping	Glucose recognition site			Biosensing	[169]
Citric acid urea	Halogen doping	D‐π‐A configuration			NIR bioimaging	[140]
p‐phenylenediamine Gd(NO_3_)_3_	Metal doping	High ^1^O_2_ QYs			Bioimaging and PDT	[299]
Fe_3_O_4_, PS‐b‐PAA CTAB	*co*‐doping	High PCE			Tumor‐specific therapy	[300]
Dopamine o‐phenylenediamine	Photosensitizer passivation	Photosensitivity	Targetable functionalization, Expand the scope of application	The preparation process is cumbersome, the purification is difficult, and the yield is low	PDT	[301]
Citric acid and diethylenetriamine	Drug passivation	Anticancer			Drug delivery and anticancer	[203]
Ammonium citrate	Charge passivation	Positive charge			Antibacterial	[217]

Non‐covalent strategies generally refers to modification methods that utilize non‐specific forces such as electrostatic interaction, complexation/chelation, hydrogen bonding, hydrophobic interactions and π‐π stacking.^[^ ^90^ ^]^ Such interactions cause minimum disturbance to the structure of the CDs.^[^ ^91^ ^]^ In contrast, covalent coupling strategy usually involves the formation of covalent bonds via the abundant surface functional groups (e.g., amines and carboxylates) on CDs.^[^ ^92^ ^]^ These functional groups allowing the chemical “linking” of CDs to other substances (e.g., inorganic/organic molecules, polymers, DNA and protein), thus allowing the preparation of composite hybrids for various applications through covalent attachments.^[^ ^93^ ^]^ This can greatly improve the compatibility in complex biological systems compared with the non‐covalent route. Further, covalent modifications can enhance the luminescence and solubility of CDs, enhancing their biological utilization.^[^ ^94^ ^]^ Covalent coupling strategies can be divided into the following categories: amidation, sulfonation, esterification, silanization and copolymerization reactions. The amidation strategy is the most commonly used method owing to the plentiful amino/carboxy groups on the surface of CDs, with the surface modification typically using 1‐ethyl‐3‐(3’‐dimethylaminopropyl) carbodiimide (EDC)/N‐Hydroxysuccinimide (NHS) or dicyclohexylcarbodiimide (DCC)/1‐hydroxybenzotriazole (HOBt) as coupling agents. Ko et al. used EDC coupling to connect CDs with biotin, then studied the in vivo permeability of the CDs‐biotin product towards BBB transit using immunohistochemical analysis of the brain (Figure 1f).^[^ ^95^ ^]^ After intraperitoneal injection, a large amount of CDs‐biotin was detected in the entire central nervous system area, including the olfactory bulb, neocortex, midbrain and cerebellum, indicating that modified CDs had the ability to penetrate the BBB in the body. In addition, Liang et al. reported a mitochondrial oxidative stress amplifier MitoCAT‐g, based on CDs‐supported gold atoms surface modified by triphenylphosphine and cinnamaldehyde. The MitoCAT‐g particles specifically targeted mitochondria and consumed mitochondrial glutathione, thereby amplifying reactive oxygen damage caused by cinnamaldehyde, ultimately resulting in cancer cell apoptosis.^[^ ^96^ ^]^

## 4 Properties

The application of nanomaterials is closely related to their properties, thus to effectively utilize CDs in biology and biomedicine deep understanding of their properties and structure‐property relationships are needed. Among the many properties of CDs, their optical properties and toxicity arguably the most important for their bioapplications. The location of CDs in organisms often employs their optical properties (especially fluorescence), whilst negligible toxicity is essential for the bioapplication of CDs. In this section, we will explore the optical properties, toxicological and the biocatalytic platforms of CDs.

### 4.1 Optical Properties

The optical properties of CDs is currently a very hot topic of research. Properties such as high QYs of fluorescence, red/NIR emission, up‐conversion photoluminescence (UCPL), NIR‐driven photothermal heating, and chiral luminescence are all being exploited in the application of CDs in biology and biomedicine.

**Figure 2:**
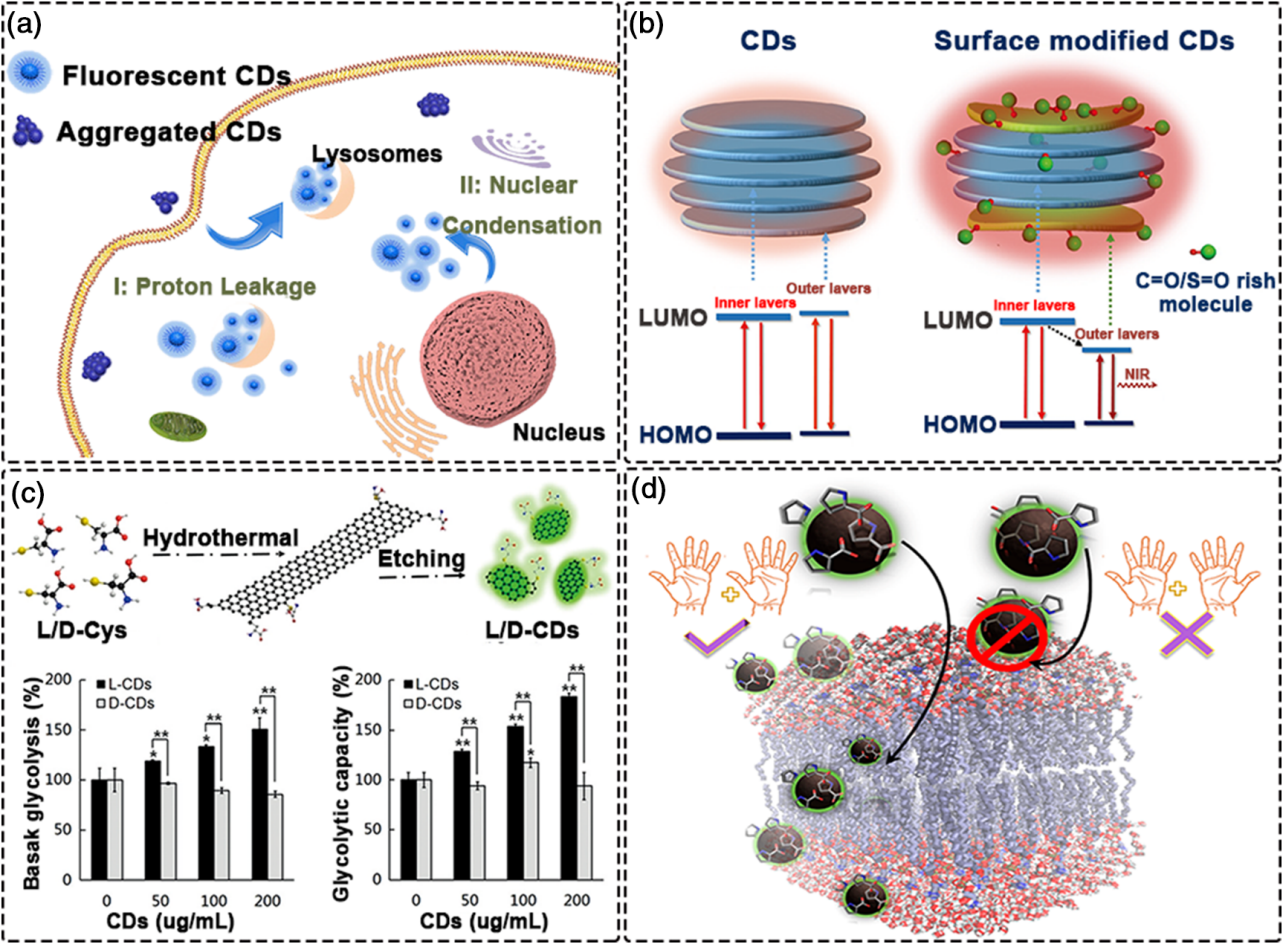
Optical properties of CDs. a) High quantum yield CDs for visualization of lysosomes. Reproduced under the terms of the CC‐BY 4.0 license.^[^ ^98^ ^]^ Copyright 2020, The Authors, published by Elsevier. b) NIR‐emitting CDs for bio‐imaging. Reproduced with permission.^[^ ^104^ ^]^ Copyright 2018, Wiley‐VCH. c) Chiral CDs for the treatment of T24 cells. Reproduced with permission.^[^ ^108^ ^]^ Copyright 2018, Wiley‐VCH. d) Chiral CDs demonstrating high selectivity for cell membranes. Reproduced with permission.^[^ ^111^ ^]^ Copyright 2018, American Chemical Society.

A high QYs is very beneficial for the in vivo tracking of CDs. Most CDs absorb in the UV‐region and emit blue‐green light, thus making detection difficult due to the poor penetration of short wavelength light in biological tissues. Blue‐green emitting CDs with high QYs can overcome this shortcoming at some extent. For CDs prepared by top‐down methods, QYs are generally low. Zhu et al used a hydrothermal method with citric acid and amines to increase the QYs of CDs to 80% for the first time.^[^ ^97^ ^]^ Subsequently, through the reaction of citric acid and *o*‐phenylenediamine, blue emission CDs with a QYs of 83% were obtained (**Figure** **2**a).^[^ ^98^ ^]^ Benefiting from the acidophilic properties of the CDs, they can be used to passively visualize lysosomes in living cells. By using Rose Bengal (RB) and branched polyethyleneimine (bPEI) as precursors, green emission CDs with QYs of 90.49% were obtained.^[^ ^99^ ^]^ Fluorescence imaging revealed that the CDs could quickly enter a cell within 10 min, almost exclusively accumulating in the lysosome and showing strong local fluorescence after 30 min of incubation. Although blue‐green emitting CDs with high QYs can be used for imaging, concomitant damage to cells and tissues results which is not ideal.

Due to minimal tissue absorption, deep tissue penetration, and low autofluorescence interference, red and NIR emitting CDs are now attracting a lot of attention.^[^ ^100^ ^]^ Various applications linked to the visualization of biological systems in vitro and in vivo have been explored. Regarding in vitro cell imaging, red/NIR‐CDs can be incubated with various cancer cells to test their potential for labeling applications. The results show that red/NIR‐CDs accumulate predominantly in the cell membrane and cytoplasm.^[^ ^101^ ^]^ Our group has successfully developed red‐emitting CDs with high QYs using o‐phenylenediamine as the precursor, them used these CDs as fluorescent probes for in vitro and in vivo imaging.^[^ ^102^ ^]^ Subsequently, we developed a variety of red‐emissive CDs based on o‐phenylenediamine, which allowed bioimaging.^[^ ^103^ ^]^ Based on a synthesis using non‐aromatic small molecule precursors (citric acid and urea) and DMSO, Qu et al. prepared CDs with a strong fluorescence emission at 715 nm which were successfully applied in bioimaging (Figure 2b).^[^ ^104^ ^]^

The chirality in CDs can have a significant impact on their end application, especially in nanotherapeutics.^[^ ^105^, ^106^ ^]^ There are many examples where chirality has a significant impact on molecular recognition in biology and medicine. Therefore, imparting chirality to nanostructures is an important direction for bioapplications. Inspired by the precise Golgi localization capabilities of galactosyltransferase and protein kinase D containing cysteine residues, Huang et al. introduced *L*‐cysteine to the surface of CDs to give chiral features (*L*‐CDs).^[^ ^107^ ^]^ The resultant *L*‐CDs exhibit excellent light stability and biocompatibility, proving suitable for long‐term in situ imaging of the Golgi. With the help of the prepared *L*‐CDs, the dynamic changes of the Golgi apparatus in the early stage of viral infection can be visualized. In addition, chiral cysteine has also been widely used as a stabilizer and chiral ligand to adjust the properties of nanomaterials. Treating human bladder cancer T24 cells with L (or D)‐cysteine derived chiral CDs, L‐CDs showed up‐regulated glycolysis, while D‐CDs offered no similar effect (Figure 2c).^[^ ^108^ ^]^ Studies have shown that chirality is transferred from surface conjugated amino acids to achiral CDs.^[^ ^109^ ^]^ This reversal is believed to be the result of highly strained intermediates produced during surface conjugation of amino acids by CDs involving carbodiimide coupling agents.^[^ ^110^ ^]^ As a result of this chirality inversion, some chiral CDs interact with the cell membrane or its mimics in a highly selective manner due to the asymmetric selectivity obtained, thereby regulating many cellular processes. For example, D‐proline with inverted chirality will preferably interact with the cell membrane or its liposome mimic (Figure 2d).^[^ ^111^ ^]^

### 4.2 Toxicity

#### 4.2.1 Evaluation

Due to their unique optical properties, large surface area and surface functionality, CDs show great potential in various bioapplications. As possible industrial and biomedical applications of CDs have been explored, biosafety issues of CDs have gradually surfaced. There are two main methods for evaluating the toxicity of CDs. One is in vitro evaluation, with this test usually involving a cell viability experiment *via* certain assays such as MTT, CCK‐8, WST‐1, etc.^[^ ^29^, ^112^, ^113^ ^]^ Cells cultured with or without CDs are regarded as the experimental and control groups, respectively. Then the toxicity of CDs can be tested by comparing these two groups. To eliminate the effect of culture dish and the selected assays, a further blank group related to external environment is usually introduced in these experiments. The second type is in vivo assessment, in which a CDs solution is directly injected into living bodies or mice and zebrafish either through the tail or a vein.^[^ ^114^, ^115^, ^116^ ^]^ Lee et al. first studied the toxicity and distribution of carboxylated CDs in mice. A certain period after administration, blood biochemical, hematological analysis and inflammatory analysis towards liver, kidney, spleen, heart or lung were performed.^[^ ^117^ ^]^ Results showed that CDs had no side effects on organisms or body tissues. The process of CDs circulation in the body can be explored through fluorescence imaging, firstly in the blood, then entering the kidneys and spleen, and finally being discharged from the body through the kidney filtration system.

#### 4.2.2 Factors

**Figure 3:**
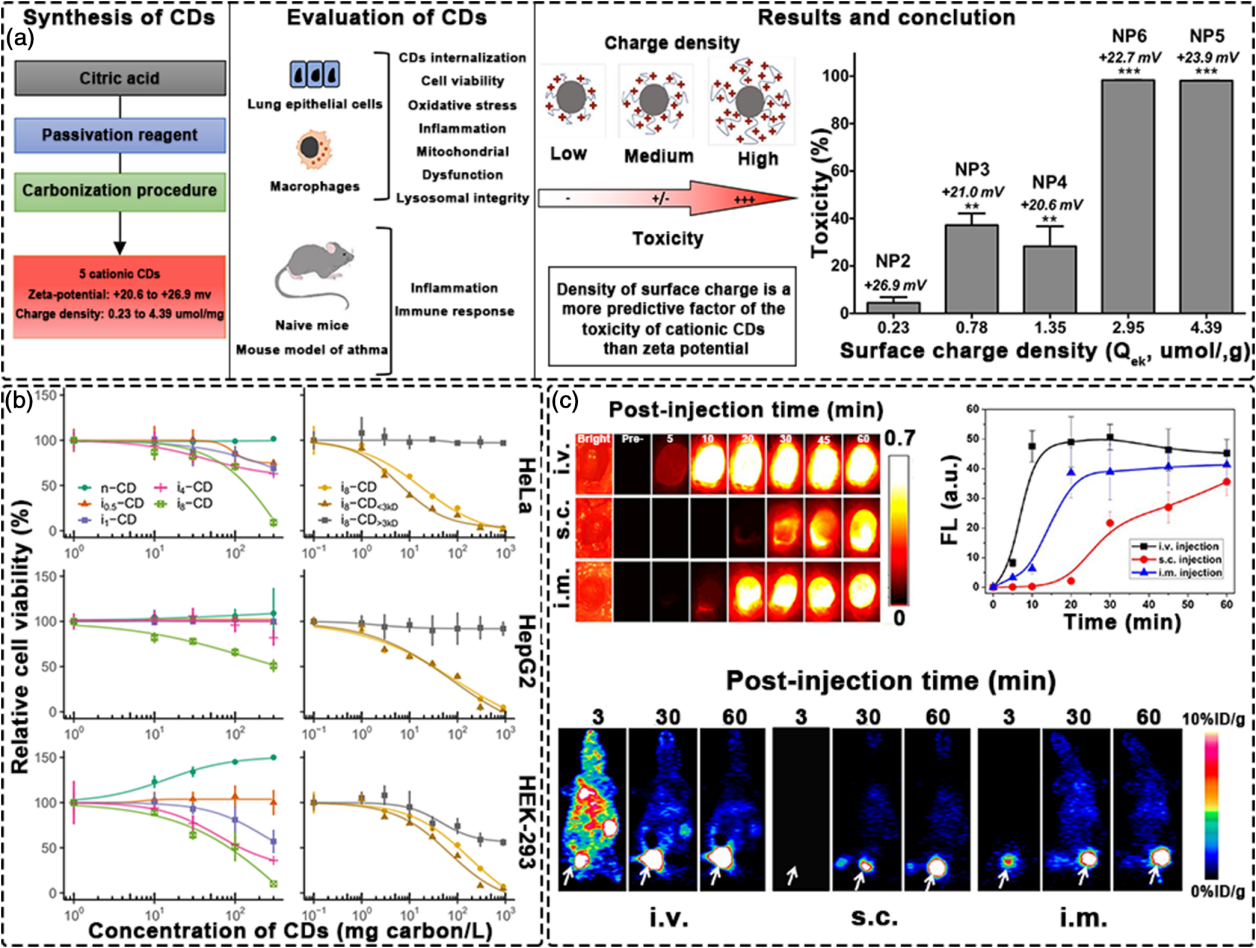
Toxicity of CDs. a) The effect of surface charge on the toxicity of CDs. Reproduced under the terms of the CC‐BY 4.0 license.^[^ ^118^ ^]^ Copyright 2020, The Authors, published by Springer Nature. b) Toxicity of CDs photolysis products. Reproduced under the terms of the CC‐BY 4.0 license.^[^ ^119^ ^]^ Copyright 2021, The Authors, published by Springer Nature. c) Metabolism of CDs in mice. Reproduced with permission.^[^ ^122^ ^]^ Copyright 2013, American Chemical Society.

Regarding toxicity studies of CDs, most reports show that CDs have very low toxicity or are non‐toxic. However, the toxicity of CDs is closely related to multiple factors including an individual CDs’ physicochemical properties to external conditions such as surface charge, photolysis, concentration, etc. Each individual type of CDs possesses its own unique properties, which in turn determines its toxicity. Among these, a positive surface charge is the main cause of the toxicity of CDs. In order to understand this more deeply, Pons et al. conducted a comprehensive study on 5 kinds of cationic CDs (CD2 to CD6). These CDs possessed a similar zeta (ζ) potential, but increasing surface charge density (*Q*_ek_ from 0.23 to 4.39 μmol g^−1^). In vivo research results show that only CD5 and CD6 with the highest *Q*_ek_ values induced significant oxidative stress, IL‐8 release, and mitochondrial dysfunction. In mice, only CD5 and CD6 induced airway inflammation. CD5 also increased the allergen‐induced immune response, airway inflammation, and mucus production (**Figure** **3**a).^[^ ^118^ ^]^ Miao et al. found that both commercial CDs purchased from Sigma‐Aldrich and CDs prepared by microwave pyrolysis of PEG and glucose decompose after being irradiated with a fluorescent lamp. Cytotoxicity was mainly associated with photolysis products in the <3 kD fraction released from the irradiated CDs (Figure 3b).^[^ ^119^ ^]^

Liu et al. used reactive red 2 (RR2), an organic pollutant, as a raw material to synthesized CDs and explore the cytotoxicity of the synthesized CDs. HeLa cells were treated with RR2 and CDs for 24 h, after which MTT analysis was performed to analyze their effects on cell viability. Compared with the control group (Ctrl), the cell viability reduced by nearly 60% when the concentration of RR2 was increased to 1000 mg L^−1^. However, CDs showed a significantly lower cytotoxicity even when the concentration of CDs was as high as 1000 mg L^−1^.^[^ ^120^ ^]^ Further, the average cell viability was greater than 70%, which means that CDs possessed only a fraction of toxicity of their precursor. In the bioapplication of CDs, concentrations as high as 1000 mg L^−1^ are never used, thus CDs are can be generally considered as low or non‐toxic. Zebrafish were then employed to further explore the toxicity of CDs in vivo. The morphological differences of zebrafish treated with different concentrations of CDs and RR2 confirmed that the toxicity of CDs was significantly reduced compared with RR2. Further, CDs (400 mg L^−1^) offered no toxic hazard to zebrafish development.

For mammals, Liu et al. used the radiolabeling method to study the biological distribution of CDs in vivo for the first time. The results indicate that CDs are gradually eliminated from mice through kidney and fecal excretion, with no obvious toxic effects of CDs on mice being observed.^[^ ^121^ ^]^ Chen et al. examined the effects of different injection routes on blood circulation, biodistribution, urine clearance and passive tumor absorption by CDs. Studies have shown that CDs can be quickly and effectively excreted from the body through urine after all three injection routes (Figure 3c).^[^ ^122^ ^]^ Low toxicity and rapid removal make CDs a viable candidate for bioapplications.

### 4.3 Biocatalyst

In addition to excellent optical properties and low toxicity, catalysis has also become an attractive field for chemists. Currently, CDs have become a mature tool and are widely used to design and develop new catalytic processes, including organic transformations, artificial photosynthesis, biocatalysis, etc.^[^ ^123^ ^]^ Among them, enzyme‐assisted biocatalytic transformation plays a key regulatory mechanism in human life activities and medical intervention, and has achieved more and more attention. By monitoring the biocatalytic transformation between different enzymes and substances, it is beneficial to prevent, identify and treat diseases. Given their unique properties, CDs are thought to enable sensitive driving of enzyme‐assisted biocatalytic transformations by detecting the expression levels of relevant metabolites.^[^ ^124^, ^125^, ^126^ ^]^ Reisner et al. found that CDs can act as an excellent light absorber in biological photosynthetic systems, using fumarate reductase to solar‐driven hydrogenation of fumarate to succinate, or hydrogenase to reduce protons to H_2_. Electrostatic interactions at the CDs‐enzyme interface are thought to be necessary for the high catalytic activity observed with modified CDs.^[^ ^127^ ^]^ The rich surface environment of CDs and their photostability and water solubility make CDs a versatile photosensitizer for oxidoreductases. Since many biochemical processes are carried out by various enzymes, the study of nanozymes that mimic complex enzymatic reactions is a formidable research goal in the field of CDs. Zhao et al. synthesized CDs with potent peroxidase‐like activity, and they could easily catalyze the formation of oxidized TMB from 3,3,5,5‐tetramethylbenzidine (TMB) in the presence of H_2_O_2_. Mo and S doping in CDs significantly enhanced their yields and might facilitate electron transfer between TMB and H_2_O_2_, thereby further enhancing the catalytic activity of CDs.^[^ ^128^ ^]^ Taking advantage of the easy‐to‐modify properties of CDs, Meng et al. composited CDs and Au nanoclusters, which maintained the superoxide dismutase (SOD)‐like activity of CDs, while improving the horseradish peroxidase activity of Au nanoclusters.^[^ ^129^ ^]^

## 5 Applications

In this section, we will discuss some important applications of CDs and CDs‐based composite in the biological and biomedical fields, including bioimaging, sensing, drug delivery, antibacterial agents, anticancer agents (photothermal therapy, photodynamic therapy, and synergistic therapy), and antiviral agents (**Table** **3**). Emphasis is placed on the relationship between the structure, properties, and applications of CDs and their composite, with a wider view towards the tailored development of CDs with specific functions.

**Table 3:** Bioapplications of CDs‐based composites

Precursors	Composite material	Advantage	Application	References
Citric acid and urea	C_3_N_4_	Promotes water‐splitting and enhances red light absorption	Bioimaging and PDT	[255]
CTAC and triethanolamine	MSN	Provide drug loading sites	Cancer cell targeting and drug delivery	[276]
Pyrene and polyethylenimine	Black phosphorus	Enhanced NIR absorption	Photothermal‐chemo combination therapy	[267]
Citric acid and urea	ZIF	Carrier for ECL labels and enhancing the ECL signals	Biosensor	[302]
Pyrene and polyethylenimine	MXene	Marrow‐bandgap nanosonosensitizer	Sonodynamic therapy	[303]
Di(Dopa‐Se)	Hydrogels	Self‐healing and adhesion properties	in vivo cancer detection	[304]

### 5.1 Bioimaging

Bioimaging enables understanding of the structure and physiological functions of cells and organisms.^[^ ^130^, ^131^, ^132^ ^]^ Therefore, it is heavily relied upon tool in the healthcare sector for the diagnosis of human diseases. Among various bioimaging methods (e.g., optical imaging, magnetic resonance imaging, computed tomography), CDs‐based optical bioimaging is attracting increasing attention. The technique utilizes optical contrast (i.e., the difference in the optical properties) between the region imaged and the surrounding background.^[^ ^133^, ^134^, ^135^ ^]^ This technique allows imaging at both the cellular and even single‐molecule level, whilst also enabling early detection, screening, diagnosis, and image‐guided treatment of life‐threatening diseases such as cancer. The imaging functionalities of CDs arise from their unique optical features (i.e., intrinsic fluorescence) or from functional agents incorporated in their core or surface.

**Figure 4:**
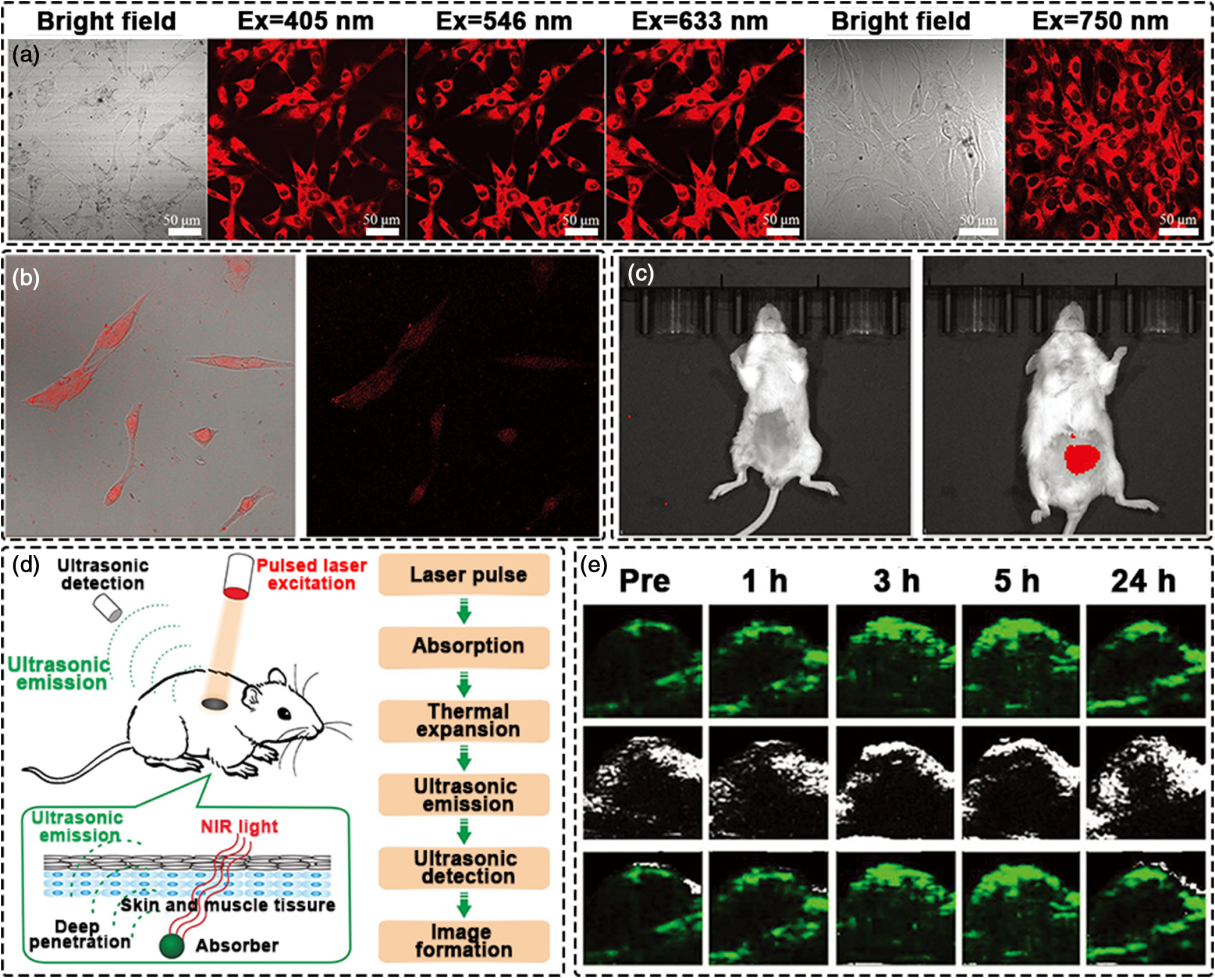
Bioimaging of CDs. a) Two‐photon CDs for bioimaging. Reproduced with permission.^[^ ^32^ ^]^ Copyright 2020, Wiley‐VCH. b) Multi‐photon CDs for bioimaging. Reproduced with permission.^[^ ^99^ ^]^ Copyright 2020, American Chemical Society. c) Two‐photon CDs in vivo imaging. Reproduced with permission.^[^ ^140^ ^]^ Copyright 2020, Wiley‐VCH. d) Schematic illustration showing the PA imaging process. Reproduced with permission.^[^ ^141^ ^]^ Copyright 2016, Ivyspring International Publisher. e) PA images in mice after i.v. injection of CDs at different time points. Reproduced with permission.^[^ ^147^ ^]^ Copyright 2019, Wiley‐VCH.

Imaging utilizing traditional UV‐absorbing and blue/green‐emitting CDs suffers from severe background interference due to the poor penetration light in biological tissues at such short‐wavelengths. Therefore, it is very important to develop CDs with red/near‐infrared emission properties for bioimaging to ensure good light penetration depth and to minimize photodamage to biological tissues.^[^ ^136^, ^137^, ^138^ ^]^ Up‐conversion photoluminescence (UCPL) can be useful in this context also. UCPL imaging (two‐photon imaging) uses near‐infrared photons to image cells or tissues, thus avoid tissue damage and autofluorescence interference commonly encountered with UV excitation. Using biomass as a carbon source, our group developed deep‐red emission CDs with excellent UPCL for simultaneous cellular and two‐photon imaging (**Figure** **4**a).^[^ ^32^ ^]^ Meanwhile, CDs with multiphoton PL emission have also been developed for cellular bioimaging.^[^ ^139^ ^]^ Two‐photon imaging using CDs can be used in vitro and in vivo (Figure 4b).^[^ ^140^ ^]^

Optical imaging is not the only imaging method that utilizes CDs. Recently, researchers have shown that CDs can also be used in photoacoustic (PA) imaging. PA is a novel bioimaging strategy that allows imaging beyond an optical diffusion limit by combing light excitation and ultrasound detection. It provides relatively deep tissue penetration with a high spatial resolution (Figure 4c).^[^ ^141^ ^]^ The contrast agent used is very important for PA imaging, because the sensitivity of the PA signal depends on light absorption and photothermal (light to heat) conversion by the contrast agent.^[^ ^142^, ^143^ ^]^ In biological systems, natural substances with special light absorption properties can be used as endogenous contrast agents for PA imaging.^[^ ^144^, ^145^, ^146^ ^]^ However, for most biological and pathological processes, endogenous contrast agents have no detectable changes and show relatively weak light absorption. Therefore, there is an urgent need to develop exogenous imaging agents with high sensitivity and specificity for PA imaging. On this basis, CDs synthesized with high absorption coefficients in red or NIR regions can produce detectable sound waves after laser irradiation, thereby allowing their action as agents for PA imaging. Owing to the excellent spatial resolution, PA imaging can provide valuable information from very small areas. For example, Qu’ group designed supra‐CDs with a strong absorption band in the visible and NIR range, which demonstrated effective NIR photothermal conversion properties. This supra‐CDs not only delivered acceptable PTT performance for tumor ablation but could also be utilized for in vivo PA imaging, thus broadening the biomedical applications of CDs (Figure 4d).^[^ ^147^ ^]^ Furthermore, excellent afterglow imaging was achieved by injecting a CDs@silica complex into the subcutaneous region of the dorsal area of mice.^[^ ^148^ ^]^ Afterglow imaging eliminates autofluorescence interference, however, it is usually difficult to prepare liquid‐phase phosphorescence CDs, which greatly limits the development of this imaging method.^[^ ^149^, ^150^ ^]^

### 5.2 Biosensing

**Figure 5:**
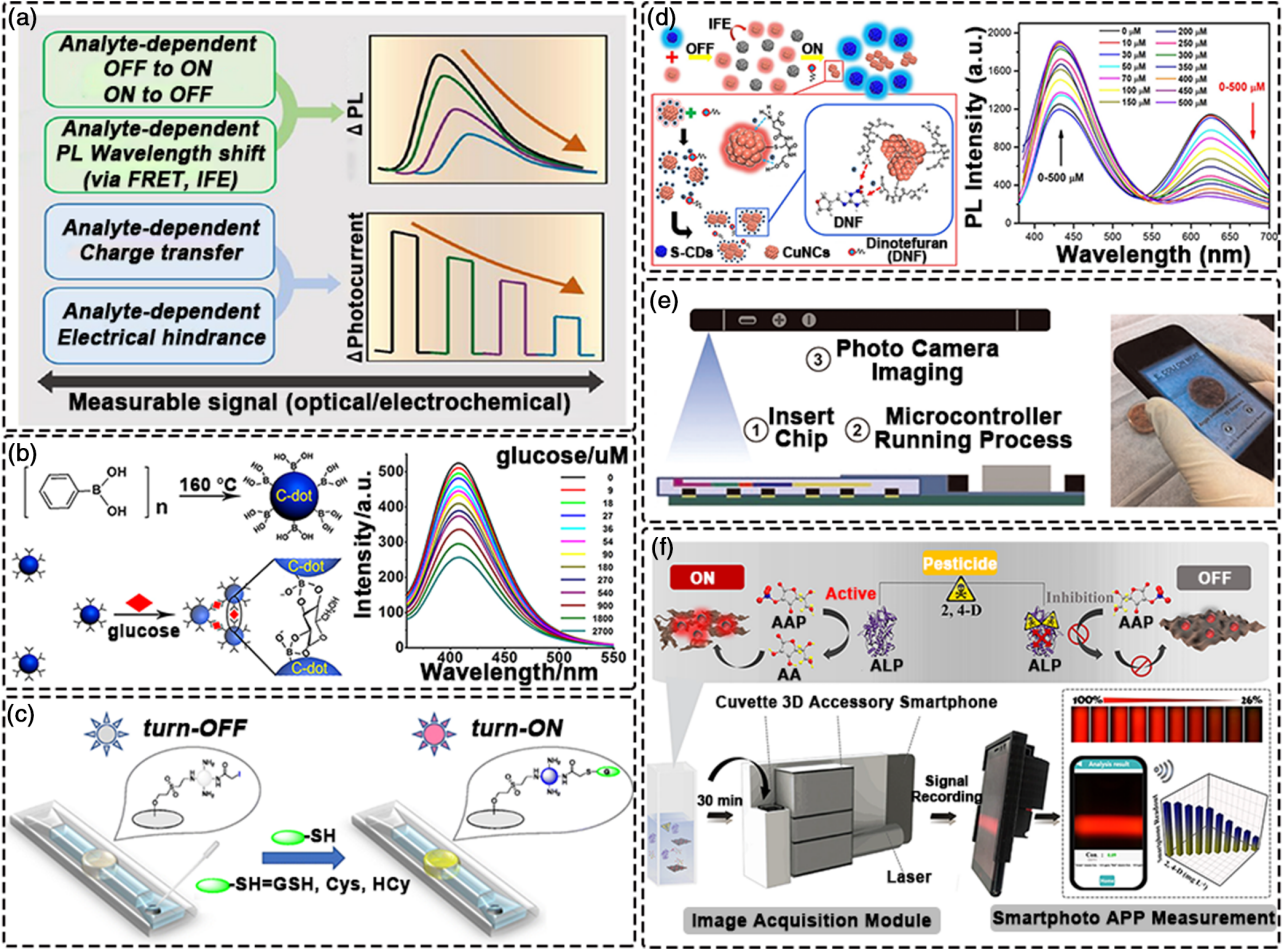
CDs for biosensing. a) Photomediated biosensing strategies using CDs’ fluorescence and photocurrent. Reproduced with permission.^[^ ^117^ ^]^ Copyright 2020, American Chemical Society. b) CDs detection of glucose by an on‐off mechanism. Reproduced with permission.^[^ ^169^ ^]^ Copyright 2014, American Chemical Society. c) CDs detection of amino acids by an off‐on mechanism. Reproduced with permission.^[^ ^171^ ^]^ Copyright 2020, Springer. d) CDs detection of DNF by a ratiometric fluorescence shift mechanism. Reproduced with permission.^[^ ^175^ ^]^ Copyright 2020, Elsevier. e) Biosensor based on a smartphone for biochemical detections. Reproduced with permission.^[^ ^177^ ^]^ Copyright 2016, Elsevier. f) Smartphone‐assisted sensing platform for the CDs‐based detection of 2,4‐dichlorophenoxyacetic acid. Reproduced with permission.^[^ ^178^ ^]^ Copyright 2020, American Chemical Society.

Biosensing is a sub‐field of analytical sensing, which places special attention on incorporating molecular biometric entities in the detection process.^[^ ^151^, ^152^, ^153^ ^]^ The excellent photophysical properties of CDs such as photoluminescence (PL) and good electrical conductivity make them valuable biosensing materials.^[^ ^154^, ^155^, ^156^ ^]^ Generally, the sensing principle of CDs is based on the specific interaction between the surface functional groups or targeting moiety and the analytes, leading to the change of CDs’ optical emission characteristics (**Figure** **5**a).^[^ ^157^, ^158^, ^159^ ^]^ In theory, any optical changes, including fluorescence intensity, colorimetric wavelength or life time can be employed as measurable signals to achieve the identification of the corresponding analyte.^[^ ^160^, ^161^, ^162^ ^]^ On this basis, CDs‐based biosensors can be classified into three categories: on‐off, off‐on, and fluorescence shift.

#### 5.2.1 On‐Off Mechanism

Among the reported sensing modes, the on‐off category (i.e., fluorescence quenching) is the most common sensing design for sensors incorporating CDs. The approach has been applied for the detection of various cations, anions and small molecules.^[^ ^163^, ^164^, ^165^ ^]^ For instance, due to the binding of metal ions by CDs, electrons are transferred from CDs to the empty d orbitals of metal ions, leading to fluorescence quenching. Several studies have identified specific surface functional groups of CDs that can coordinate metal ions (such as Cu^2+^ and Fe^3+^) in biological systems.^[^ ^166^, ^167^ ^]^ For example, CDs with abundant phenolic hydroxyl groups exhibited a good binding affinity towards Fe^3+^, with complexation leading to splitting of the d orbitals of Fe^3+^. Electrons in the photoexcited CDs were partially transferred to Fe^3+^, resulting in fluorescence quenching.^[^ ^168^ ^]^ Further, some biologically active molecules, such as glucose, can specifically bind to CDs prepared using phenylboronic acid, again achieving “on‐off” detection of glucose (Figure 5b).^[^ ^169^ ^]^ In this process, the *cis*‐diols group of glucose react with boronic acid groups on the CDs’ surface, resulting in fluorescence quenching through aggregation states.

#### 5.2.2 Off‐On Mechanism

The “off‐on” mechanism utilizes an increase in the fluorescence intensity of CDs in the presence of an analyte. In this strategy, the analyte concentration can be measured by returning the quenched CDs to the emission state. A more favorable interaction/reaction with the analyte disrupts the interaction between the CDs and the quencher, hence the CDs regain their characteristic fluorescence. Generally, there are two routes for such “off‐on” detection. One is the removal of quencher from the surface of CDs, another is the release or detachment of CDs from the quencher. In the first case, “cation ion‐mediated” or “anion ion‐mediated” strategies are usually employed. For instance, after fluorescence quenching, the emissive properties of the CDs can be recovered by addition of pyrophosphate (PPi), enabling detection of PPi and fluorescence imaging of intracellular PPi via “off‐on” detection of PPi.^[^ ^170^ ^]^ Moreover, since iodine can undergo a substitution reaction with biological thiols, CDs modified with iodine can be used for the fluorescence turn‐on measurement of biological thiols (GSH, Cys, and Hcy) (Figure 5c).^[^ ^171^ ^]^ For the second strategy, nanomaterials such as layered transition‐metal oxides or disulphides (e.g., MnO_2_ and MoS_2_), graphene oxide (GO) and metal nanoparticles (NPs) (e.g., Ag and Au NPs) are generally utilized as quenchers. On this basis, the addition of analytes releases CDs from the nanomaterials enabling fluorescence recovery.^[^ ^172^, ^173^, ^174^ ^]^

#### 5.2.3 Fluorescence Shift Mechanism

Unlike the “on‐off” sensing strategy that quenches the emission of a single fluorophore, the partial spectral overlap between donor emission and acceptor absorption allows the ratiometric detection of analytes. The concentration of the analyte can be quantified in a ratio by comparing the intensities at two emission wavelengths. Through the self‐calibration of the two emission bands, the ratio change provides a more accurate and sensitive signal output compared with measurements based on a single fluorescence maximum. A dual emission ratiometric fluorescent probe based on a hybrid composite involving S‐doped CDs and copper nanoclusters (CuNCs), exploiting the inner filter effect (IFE) between S‐CDs and CuNCs, was successfully applied in the selective ratiometric detection of dinotefuran (DNF) (Figure 5d).^[^ ^175^ ^]^

Currently, researchers are paying more attention to the real‐time monitoring of samples, whilst seeking to avoid the time‐consuming and cumbersome sample preparation routines commonly associated with conventional analytical instruments.^[^ ^176^ ^]^ With an increasing demand for rapid detection speeds and high sensitivity, smartphones are now widely used for real‐time monitoring as analyzers due to their portability and ease of operation (Figure 5e).^[^ ^177^ ^]^ For example, Lu et al. developed a light‐sensing platform based on CDs, which integrated smartphone applications to detect 2,4‐dichlorophenoxyacetic acid in real‐time (Figure 5f).^[^ ^178^ ^]^ This portable platform highlights exciting new horizons for on‐site monitoring, with a widespread future application of CDs and smartphones in the field of bioanalysis being likely soon.

### 5.3 Drug Delivery

**Figure 6:**
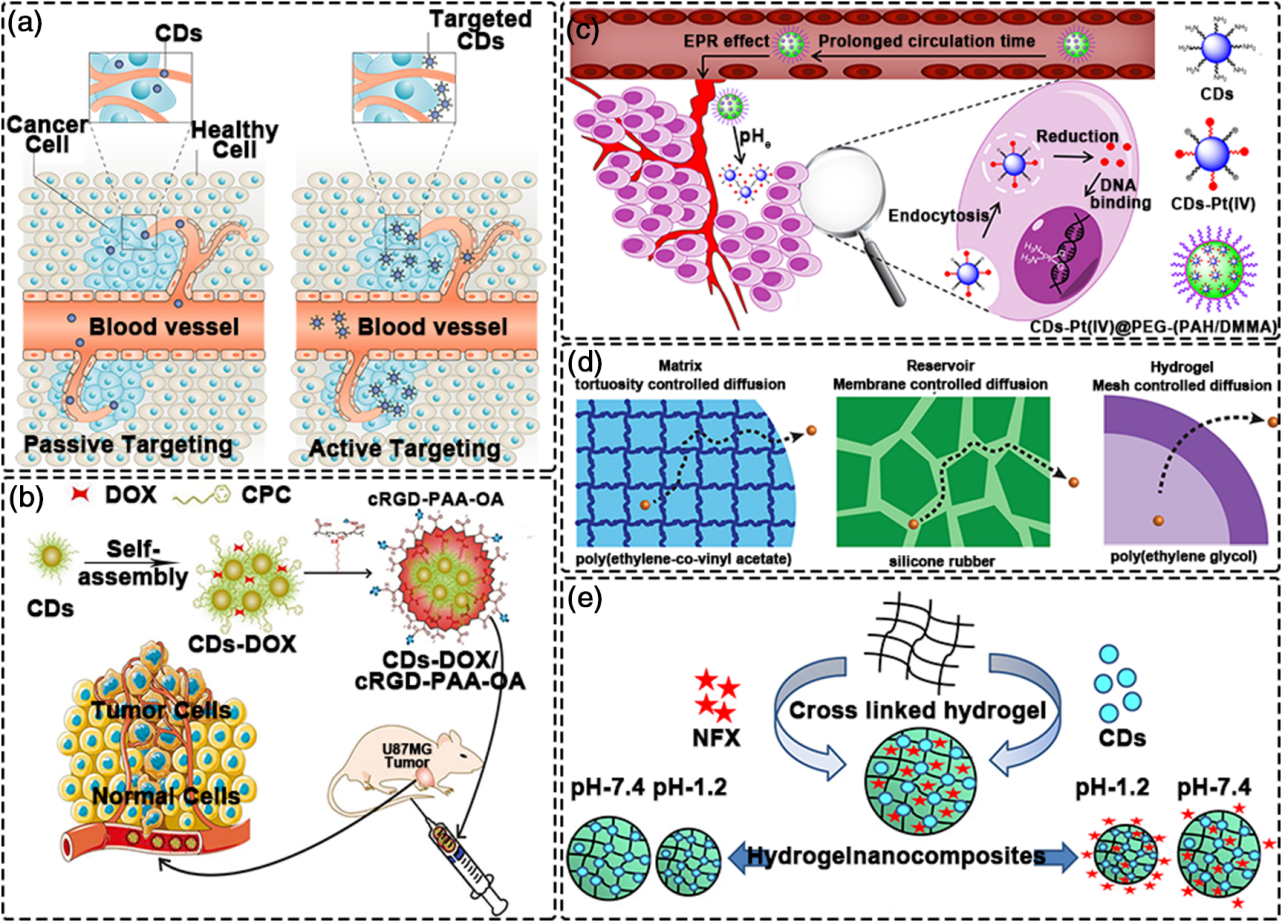
Drug delivery with CDs. a) CDs utilizing the EPR effect to enter a lesion. Reproduced with permission.^[^ ^179^ ^]^ Copyright 2019, Springer Nature. b) CDs as a delivery platform for DOX. Reproduced with permission.^[^ ^195^ ^]^ Copyright 2018, Wiley‐VCH. c) CDs being used to deliver cisplatin. Reproduced with permission.^[^ ^203^ ^]^ Copyright 2016, American Chemical Society. d) Mechanisms of controlled drug release. Reproduced with permission.^[^ ^208^ ^]^ Copyright 2016, American Chemical Society. e) CDs‐containing hydrogel for the controlled release NFX. Reproduced with permission.^[^ ^209^ ^]^ Copyright 2019, Springer.

Nanomaterial‐based drug delivery involves the incorporation of drugs into nanoparticle carriers through encapsulation, adsorption or binding, thus allowing safe and stable administration in the body (**Figure** **6**a).^[^ ^179^ ^]^ To date, various kinds of anticancer or antibacterial drugs have been successfully delivered.^[^ ^180^, ^181^, ^182^ ^]^ CDs, due to their easy modification, represent effective carriers for the targeted delivery of anti‐cancer drugs (e.g., doxorubicin (DOX), cisplatin, paclitaxel, docetaxel, camptothecin, daunorubicin etc.) for cancer therapy.^[^ ^183^, ^184^, ^185^ ^]^ Most of the CDs@drug complexes have cleavable chemical bonds, allowing the targeted release of drugs under the acidic environment of tumor sites or in response to other stimuli.^[^ ^186^, ^187^, ^188^ ^]^ In addition to enhancing the delivery and controlled release at the target site, CDs can help overcome the drug resistance of cancer cells or other diseased cells.

Doxorubicin is a widely used chemotherapy drug, representing one of the most effective chemotherapy drugs currently approved by the USA. FDA. DOX can be combined with DNA‐related enzymes of cancer cells to intercalate DNA base pairs to inhibit their synthesis and replication, thus representing a common first‐line therapy for many cancers.^[^ ^189^, ^190^, ^191^ ^]^ In recent years, the use of biocompatible CDs for the targeted release of DOX in the tumor microenvironment has attracted widespread attention. Most of these pioneering works have focused on enhancing the EPR (Enhanced Penetration and Retention) effect to provide higher tumor accumulation and increase the drug loading capacity. By constructing CDs with a hydrophobic carbon core and a hydrophilic shell, composites of CDs and DOX have been assembled that show significant tumor targeting and enhanced anti‐tumor efficacy, with reduced adverse reactions.^[^ ^192^, ^193^, ^194^ ^]^ It is difficult for conventional CDs to combine with DOX, thus surface modification of CDs is required. The microenvironment of the tumor site is acidic, necessitating careful consideration of the chargeability of CDs@DOX systems. CDs@DOX with selective tumor targeting of human glioblastoma cell line (U87MG) was achieved by surface charge modification with an overcoat of cRGD and octylamine‐modified polyacrylic acid (cRGD‐PAA‐OA). Meanwhile, in in vitro drug release experiments, CDs‐based composite materials showed a good release profile of DOX in time (Figure 6b).^[^ ^195^ ^]^

In addition to DOX, cisplatin (Pt(IV)) is often used to treat many types of cancer.^[^ ^196^, ^197^, ^198^ ^]^ However, inherent and/or acquired drug resistance, as well as the terrible side effects, usually hinder the efficiency of cis‐platin‐based cancer treatments. Therefore, it is important to develop drug targeting systems with high site specificity, whilst also controlling the release‐rate of drug. Due to their small size and easy functionalization, CDs represent a good carrier for such drugs. Combining CDs with Pt(IV)‐base drugs requires careful consideration. Appropriate surface functionalization allows CDs to fix Pt(IV)‐based drugs.^[^ ^199^, ^200^, ^201^ ^]^ Among surface functionalization approaches, PEGylation is commonly used.^[^ ^202^ ^]^ The CDs platform (CDs‐Pt (IV)@PEG‐(PAH/DMMA)) based on cisplatin can promote the release of positively charged CDs‐Pt (IV) via an electrostatic repulsion mechanism. In vivo experiments verified the CD's high tumor suppressive efficacy and low side effects, demonstrating the potential of CDs as a smart nanocarrier if Pt(IV)‐based drugs for enhanced therapeutic effects (Figure 6c).^[^ ^203^ ^]^

Aside from the amount of drug loaded, controlled release and slow release of drugs are still issues that need to be addressed regarding CDs‐based systems.^[^ ^204^ ^]^ An ideal sustained‐release system should be able to deliver drugs that are quickly cleared or degraded when administered alone.^[^ ^205^ ^]^ Further, it is also necessary to protect the drug from being destroyed during the release period and to release the therapeutic agent predictably during the treatment period.^[^ ^206^, ^207^ ^]^ The aforementioned reports demonstrate that drugs can be loaded on CDs through various strategies, then released or detached from the CDs in a controlled or stimulated manner. Polymer matrices, hydrogel networks or mesoporous silica are generally adopted as part of CDs‐based controlled release systems, acting to increase local therapeutic effects and minimize side effects (Figure 6d).^[^ ^208^ ^]^ In order to better design and fabricate CDs‐based drug delivery systems with desired properties, mechanisms of controlled release should be fully understood. At present, controlled release mechanisms can generally be divided into three categories. 1) Therapeutic molecules diffuse through a tortuous network of interconnected pores formed during the phase separation of the drug/excipient and the polymer; 2) Reservoir controlled release systems restricts the release of trapped therapeutic agent through a membrane that regulates the rate at which the therapeutic agent diffuses out of the reservoir; and 3) The rate of drug release from hydrogel‐based release systems is controlled by the mesh size of the swelling polymer network. As an example of the latter, a pH‐responsive biodegradable hydrogel nanocomposite was prepared based on an agarose‐poly(vinyl alcohol) copolymer and CDs as the cross‐linker. The release of antibacterial drug norfloxacin (NFX) from the hydrogel nanocomposite followed zero‐order kinetics, with a Korsemeyer‐Peppas model confirming that the release of NFX occurred through the erosion of the hydrogel nanocomposite (Figure 6e).^[^ ^209^ ^]^

### 5.4 Antibacterial

**Figure 7:**
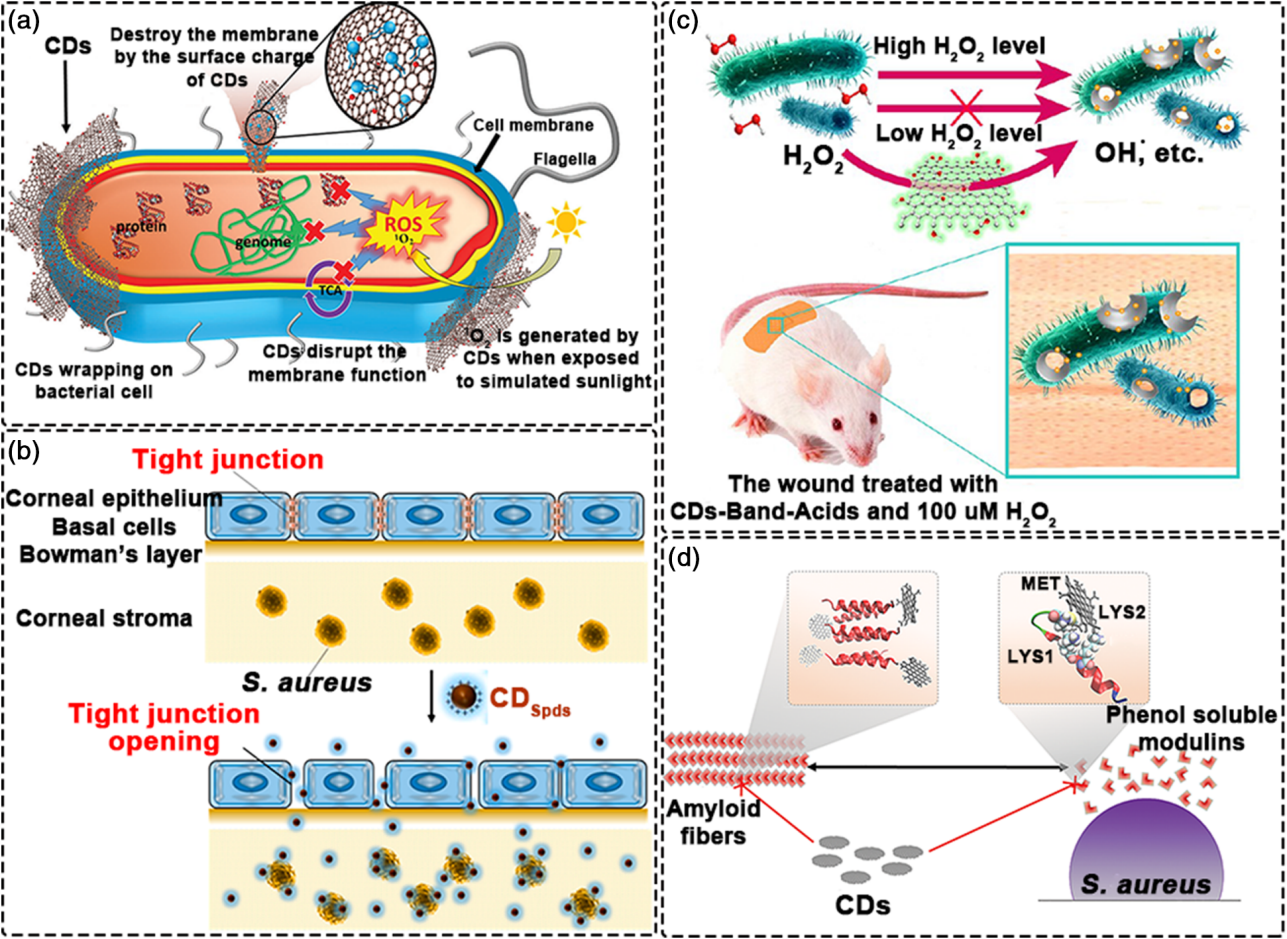
Antibacterial applications of CDs. a) Antimicrobial activity mechanism of CDs through various physical and chemical interactions. Reproduced with permission.^[^ ^213^ ^]^ Copyright 2019, Royal Society of Chemistry. b) Antibacterial activity using the surface charge on CDs. Reproduced with permission.^[^ ^220^ ^]^ Copyright 2017, American Chemical Society. c) Antibacterial activity using ROS produced by CDs. Reproduced with permission.^[^ ^227^ ^]^ Copyright 2014, American Chemical Society. d) Antibacterial activity using the anti‐biofilm properties of CDs. Reproduced with permission.^[^ ^238^ ^]^ Copyright 2019, American Chemical Society.

Owing to the increasing resistance of bacteria to conventional antibacterial agents, the discovery of new antibacterial agents with superior performance and specificity is becoming essential.^[^ ^210^, ^211^, ^212^ ^]^ As a substitute for antibiotics, antimicrobial nanomaterials have attracted great attention in recent years due to their superior performance and unique mechanisms of action on microorganisms. CDs show enormous potential as antibacterial agents due to their easy functionalization with antibacterial functional groups or controllable surface charge (**Figure** **7**a).^[^ ^213^, ^214^, ^215^ ^]^ By controlling the surface charge and functional properties, the affinity, specificity and antibacterial properties of the CDs can be precisely controlled.

CDs typically find use as antibacterial agents due to their surface charge. Qu et al. utilized different precursors to prepare three types of CDs with positive, negative and uncharged surfaces.^[^ ^216^ ^]^ These CDs were then applied for the inhibition of *E. coli* (a Gram‐negative bacterium). Positively charged CDs were shown to destroy the bacterial cell plasma membrane, resulting in significant bactericidal activity. Most CDs are prepared by the bottom‐up method have a surface rich in hydroxyl and carboxyl groups (as a result, they generally have neutral or negatively charged surfaces at neutral pH). Direct synthesis of positively charged CDs is more challenging. Therefore, post‐modification methods are generally needed to modify the positively charged side chains such as amines, imines and quaternary ammonium salts on the surface of CDs.^[^ ^217^, ^218^, ^219^ ^]^ That said, some works have been successful in directly synthesizing cationic CDs. Huang et.al. developed super‐cationic CDs by pyrolysis of biogenic positively charged spermidine powder through a simple dry heating treatment (Figure 7b).^[^ ^220^ ^]^ The CDs produced had good antibacterial activity against a variety of gram‐negative bacteria, causing serious damage to the bacterial membrane owing to their high positive charge (*ζ*‐potential ≈ +45 mV). Local ocular administration in rabbits induced the opening of tight junctions of corneal epithelial cells, resulting in a good antibacterial effect against Staphylococcus aureus. In addition, Ag, ZnO and other nanoparticles that have antibacterial activity can also be employed alongside CDs to deliver broad spectrum antibacterial activity.^[^ ^221^, ^222^, ^223^ ^]^

As is well known, reactive oxygen species (ROS) (e.g., superoxide anion, H_2_O_2_, •OH, ^1^O_2_) can kill specific cells and tissues. Therefore, the production of ROS is a further strategy for achieving potent antibacterial effects.^[^ ^224^, ^225^, ^226^ ^]^ Qu et al. developed CDs with the catalase‐like activity, capable of catalyzing the decomposition of H_2_O_2_ to produce •OH. Taking advantages of the potent antibacterial activity of •OH, the toxicity of H_2_O_2_ at high levels towards normal cells in the wound could be avoided (Figure 7c).^[^ ^227^ ^]^ Subsequent in vitro experiments show that CDs greatly enhanced the antibacterial activity of H_2_O_2_, resulting in broad‐spectrum antibacterial activity against both Gram‐negative and Gram‐positive bacteria. In addition to the endogenous antibacterial activity, light could be used to further excite CDs to produce ROS and heat.^[^ ^228^, ^229^, ^230^ ^]^ Some CDs were shown to produce free radicals after exposure to blue light (via electron‐hole pair generation and reactions to create ROS). which in turn increased the level of intracellular ROS and reduced cell viability.^[^ ^231^ ^]^ Such synergistic strategies inform the future development and safe application of CDs‐based systems for potential antimicrobial applications.

The efficacy of fungicides is often reduced by biofilms on the surface of bacteria.^[^ ^232^, ^233^, ^234^ ^]^ The protective network created by biofilms around the bacterial community effectively blocks the entry of antibacterial agents. Many anti‐biofilm agents, based on small molecules, are rapidly degraded by bacteria which reduces their efficacy. Further, the biological toxicity of many anti‐biofilm agents creates additional obstacles to their practicality. Owing to their small size, CDs can effectively penetrate into biofilms of gram‐negative and gram‐positive bacteria, typically selectively interacting with gram‐positive bacteria through electrostatic and hydrophobic interactions, finally eradicating the biofilm of gram‐positive bacteria.^[^ ^235^, ^236^, ^237^ ^]^ To investigate the mechanism of CDs against biofilms, VanEpps et al. employed a combination of theory and experiment. Supramolecular complexes were synthesized using CDs and phenol‐soluble modulator proteins, which interfered with the self‐assembly of amyloid fibers, thereby effectively dispersing mature amyloid‐rich *S. aureus* biofilms (Figure 7d).^[^ ^238^ ^]^ Modeling results show that the CDs docked near the N‐terminus of certain peptides and changed the secondary structure of the phenol‐soluble modulator protein, thereby destroying their fibrillation.

### 5.5 Anticancer

Aside from the obvious application as a drug carrier for chemotherapy, CDs can also be employed as phototherapy agents (including photodynamic therapy (PDT), photothermal therapy (PTT) or combined PDT/PTT).^[^ ^239^, ^240^ ^]^ These properties originate from the excellent optical properties of CDs including photostability and intense light absorption over a wide wavelength range.^[^ ^241^ ^]^ As the name suggests, phototherapies harness light to treat diseases, representing a promising noninvasive treatment for cancers.^[^ ^242^ ^]^

#### 5.5.1 Photodynamic Therapy

**Figure 8:**
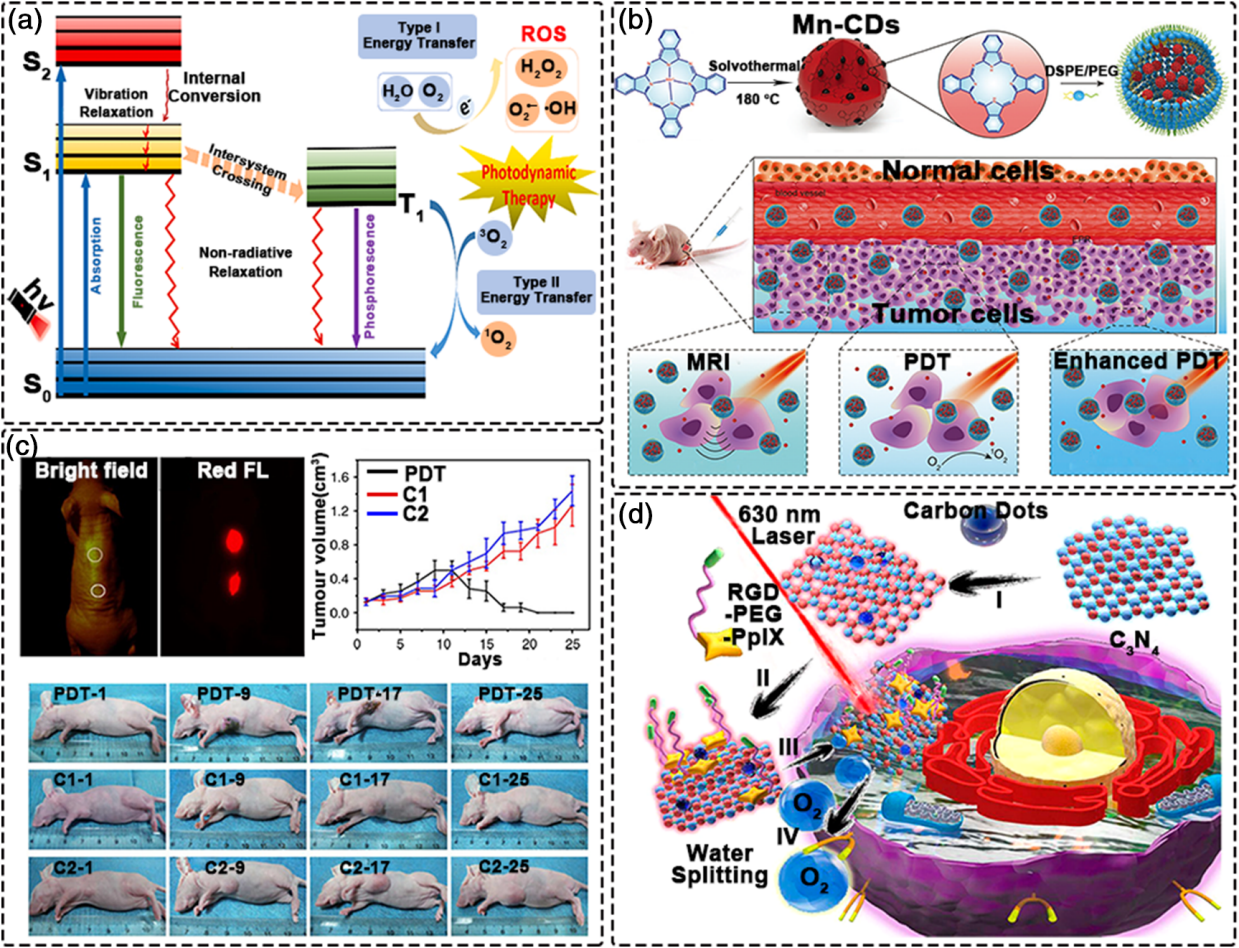
Photodynamic therapy using CDs. a) Schematic illustration of PDT mechanisms. Reproduced with permission.^[^ ^247^ ^]^ Copyright 2021, Elsevier. b) CDs directly used as photosensitizers from manganese (II) phthalocyanine for PDT. Reproduced with permission.^[^ ^248^ ^]^ Copyright 2018, Wiley‐VCH. c) CDs directly used as photosensitizers from polythiophene for PDT. Reproduced with permission.^[^ ^249^ ^]^ Copyright 2014, Springer Nature. d) CDs combined with a photosensitizer for PDT. Reproduced with permission.^[^ ^255^ ^]^ Copyright 2016, American Chemical Society.

PDT involves three key elements: A photosensitizer (PS), light and oxygen.^[^ ^243^, ^244^, ^245^ ^]^ Firstly, the PS absorbs energy from light and transitions from the ground electronic state (^0^PS) into the excited singlet state (^1^PS*). The excited PS (^1^PS*) then decays back to ^0^PS via fluorescence, or instead ^1^PS* can undergo an intersystem crossing (ISC) process to form a triplet state (^3^PS*). Next, ^3^PS* facilitates energy‐transfer or electron/proton processes that convert nearby oxygen molecules into cytotoxic ROS species, leading to cell apoptosis and necrosis.^[^ ^246^ ^]^ PDT processes are classified according to the types of electronic transitions that occur after light excitation and also the type of ROS produced. ^3^PS* can decay back to the ground state (^0^PS) without light emission (phosphorescence). During this process, the excited‐state energy can be transferred to O_2_ to form ^1^O_2_. This reaction is called a type II process (i.e., energy‐transfer process). For the type I process, PS directly reacts with substrates such as organic molecules or cell membrane to transfer protons or electrons to form free radicals (electron/proton process), and then reacts with O_2_ to produce superoxide or peroxide and destroy malignant cells through oxidation (**Figure** **8**a).^[^ ^247^ ^]^

Firstly, CDs can act directly as photosensitizers to generate ROS under light irradiation. Wang et al. successfully prepared magnetic CDs using manganese (II) phthalocyanine as the precursor (Figure 8b).^[^ ^248^ ^]^ The CDs retained the ability of phthalocyanine to produce ^1^O_2_ under 635 nm laser irradiation, producing ^1^O_2_ very efficiently (quantum yield: 0.40). Further, the CDs effectively catalyzed the decomposition of H_2_O_2_ to produce O_2_ in the acidic tumor microenvironment, thereby overcoming the hypoxia problem in solid tumors and promoting the PDT effect. Researchers from the same group also used polythiophene as a carbon source to synthesize CDs. Though polythiophene itself has almost no ability to generate ROS, the prepared CDs could generate ^1^O_2_ with a quantum yield of 1.3 through a polymorphic sensitization process. These studies demonstrate that CDs are effective PDT agents for cancer treatment (Figure 8c).^[^ ^249^ ^]^

In most cases, it is difficult for individual CDs to generate enough ROS for effective treatments. Thus, combining CDs with traditional photosensitizers is an effective strategy to improve PDT efficiency.^[^ ^250^, ^251^, ^252^ ^]^ In addition, the water solubility provided by CDs allows CDs@PS to be used in biological applications where the PS alone cannot due to its low solubility. Many attempts have been made to functionalize CDs with molecular dyes (chlorin, porphyrin, and BODIPY derivatives).^[^ ^253^ ^]^ The Förster resonance energy transfer (FRET) between CDs and dye allows synergistic photoactivation. Chen et al. constructed a FRET system by functionalizing the surface of CDs with Ce6 (a clinically‐used PS). Compared with free Ce6, the CDs@Ce6 system exhibited a faster ROS generation rate, lower darkness toxicity, and higher phototoxicity.^[^ ^254^ ^]^ Due to the increased permeability and EPR effect brought by the small size of CDs, the CDs@Ce6 system effectively accumulated at the tumor site for enhanced PDT. A polymer containing a protoporphyrin photosensitizer, polyethylene glycol segment and targeting Arg‐Gly‐Asp motif was introduced into C_3_N_4_ nanoparticles doped with CDs (PCCN). The use of PCCN increased the concentration of O_2_ in cells and improved the production of ROS in hypoxic and normoxic environments under light irradiation (Figure 8d).^[^ ^255^ ^]^ Cell viability assays showed that PCCN completely reversed the PDT resistance caused by hypoxia and showed satisfactory cancer cell growth inhibition at a concentration of 1% O_2_. In order to overcome the drawbacks of short penetration depth of many blue‐green emitting CDs, Jiang et al. developed red‐emitting two‐photon CDs for nucleolus‐targeted therapy with excellent ^1^O_2_ production under 638 nm laser irradiation. The ^1^O_2_ cleaved RNA strands for specific self‐targeting of the nucleolus during PDT.^[^ ^256^ ^]^

#### 5.5.2 Photothermal Therapy

**Figure 9:**
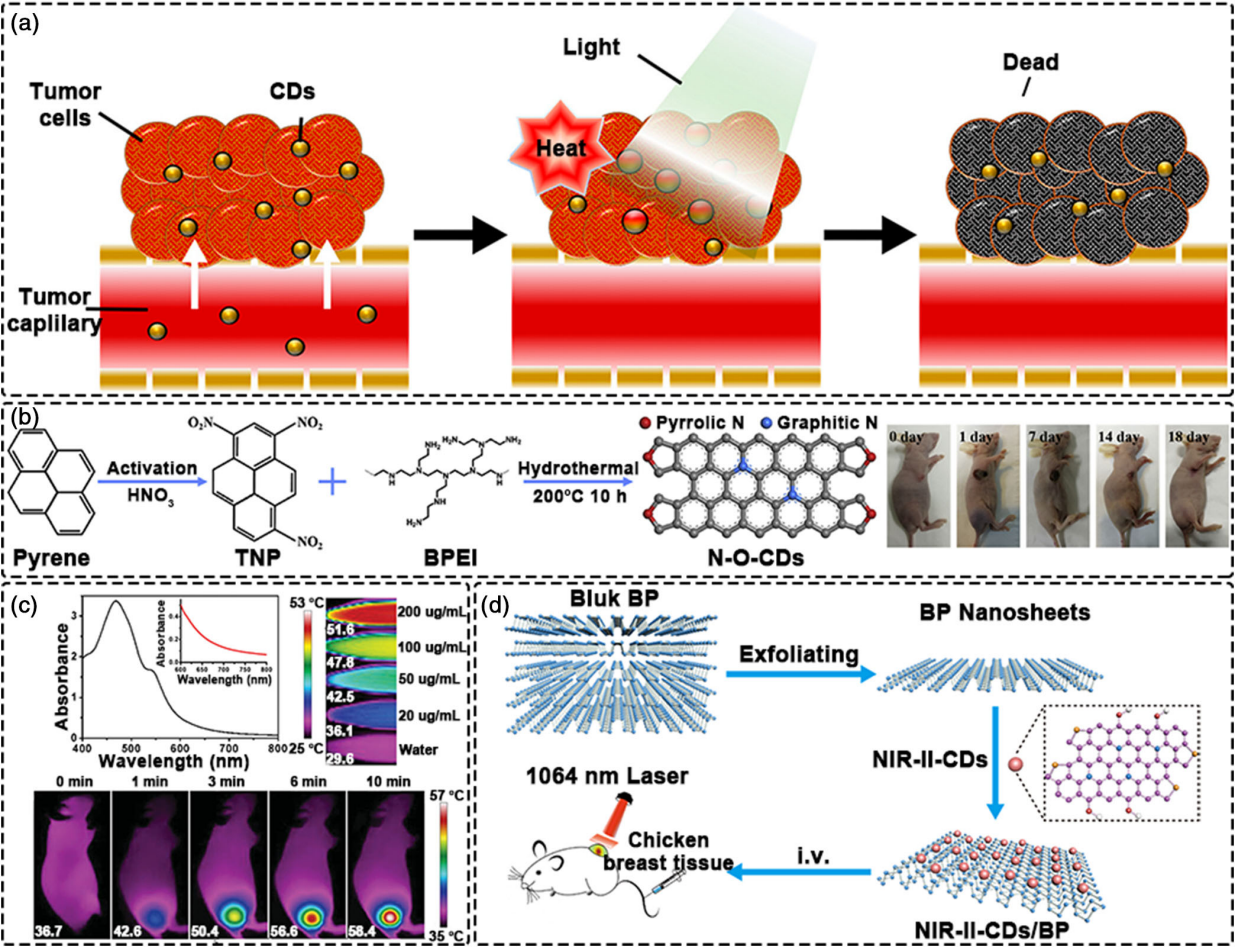
Photothermal therapy using CDs. a) Schematic illustration of PTT mechanisms. Reproduced under the terms of the CC‐BY 4.0 license.^[^ ^257^ ^]^ Copyright 2018, The Authors, published by MDPI. b) CDs directly used as photothermal agent by N doping for PTT. Reproduced with permission.^[^ ^263^ ^]^ Copyright 2018, Elsevier. c) CDs from polythiophene phenylpropionic acid directly used as photosensitizers for PTT. Reproduced with permission.^[^ ^265^ ^]^ Copyright 2015, Wiley‐VCH. d) CDs combined with photothermal agents for PTT. Reproduced with permission.^[^ ^267^ ^]^ Copyright 2019, American Chemical Society.

Photothermal therapy (PTT) is a kind of phototherapy that does not rely on oxygen. It depends on ablative agents (such as nanomaterials with photothermal effect) to convert light into heat (**Figure** **9**a).^[^ ^257^ ^]^ Elevated temperatures kill cancer cells while minimizing obvious side effects on normal cells.^[^ ^258^, ^259^, ^260^ ^]^ This approach exploits the fact that normal cells have higher heat tolerance than cancer cells.^[^ ^261^, ^262^ ^]^ Ideally, a photothermal agent should satisfy all of the following criteria: 1) Strong absorption in the NIR region with a low fluorescence QYs and singlet oxygen production rate, thereby maximizing the conversion of absorbed photons into heat; 2) non‐toxic properties without light irradiation, but selectively kills cancer cells under red light or NIR irradiation; and 3) easy to prepare and modify. CDs have the potential to meet all of these requirements, and therefore are considered very promising agents for PTT.

To improve NIR absorption efficiency of CDs, it is necessary to start with the CDs structure. From the perspective of carbon core, it is necessary to control the content of graphitic N inside the carbon skeleton. The emission wavelength of CDs gradually redshifts with the increase of graphitic N content. CDs prepared from 1,3,6‐trinitropyrene (TNP) and branched polyethyleneimine (BPEI) as carbon sources have high light stability and excellent biocompatibility (Figure 9b).^[^ ^263^ ^]^ Under low power density light (0.8 W cm^−2^) irradiation, a high photothermal conversion efficiency (PCE) (38.3%) was achieved, which is beneficial to PTT in vitro and in vivo. The absorbance at 808 and 1064 nm were proportional to the graphitic N content, with the PTT temperature being controlled by the laser intensity at these wavelengths. Hence, as the graphitic N content increases, the PCE is significantly improved. In the NIR‐II window, an ultra‐high PCE of 81.3% was achieved.^[^ ^264^ ^]^ In addition, in lesions with a thickness >10 mm, a 1064 nm laser resulted in complete tumor ablation at a low laser power density.

Most CDs prepared to date have relatively low NIR absorption. Some CDs can selectively kill cancer cells effectively under red irradiation. Wang et al. used polythiophene phenylpropionic acid (PPA) as a precursor to prepare CDs (Figure 9c).^[^ ^265^ ^]^ The as‐prepared CDs have a wide absorption range (400–800 nm), with emission peaks in the range of 500–800 nm. These CDs possessed a PCE of 38.5% under 671 nm laser irradiation. The CDs prepared by the solvothermal reaction of citric acid and urea have a maximum absorption at 600 nm, delivering a high PCE (59.19%) under 655 nm laser irradiation.^[^ ^266^ ^]^ However, NIR‐driven PEC is preferred to red‐driven PEC, since few biological substances absorb in the NIR region.

A further approach in the development of PEC agents is to introduce other photothermal agents, such as metal or non‐metal nanoparticles with good PCE response, on the surface of CDs to give impart new functions and achieve 1 + 1 > 2 effects. Layered black phosphorous (BP) nanosheets have been widely used in PTT due to their excellent photothermal effect and large surface area, but their easy oxidation under environmental conditions limits their large‐scale application (Figure 9d).^[^ ^267^ ^]^ The addition of CDs can not only protect BP from water and oxygen, but also enhance the photothermal performance of BP nanosheets, thus achieving a high PCE in the NIR‐I (77.3%) and NIR‐II (61.4%) windows. These PCEs were significantly higher than those of the original BP nanosheets (49.5% and 28.4% at 808 and 1064 nm, respectively). CuS also has very good absorption in the NIR region owing to electronic transitions. Accordingly, it also represents a very effective photothermal conversion material. Combining CDs with CuS nanoparticles (CuS‐CDs), excellent PCE was achieved under 808 nm laser irradiation. After exposure to a 808 nm laser (0.5 W cm^−2^) for 7 min, the temperature of a CuS‐CDs dispersion (100 μg mL^−1^) reached 65.6 °C, whereas the temperature of a CuS dispersion and a water control reached only 47.5 and 25.8 °C, respectively. The PCE of CuS‐CDs was 39.7%, 1.9 times higher than that of CuS (21%).^[^ ^268^ ^]^

#### 5.5.3 Synergistic Treatment

With a view towards more effective cancer treatment, collaborative treatments including imaging‐guided drug delivery, phototherapy and PDT‐PTT combination therapy are becoming increasingly attractive.^[^ ^269^, ^270^, ^271^ ^]^

**Figure 10:**
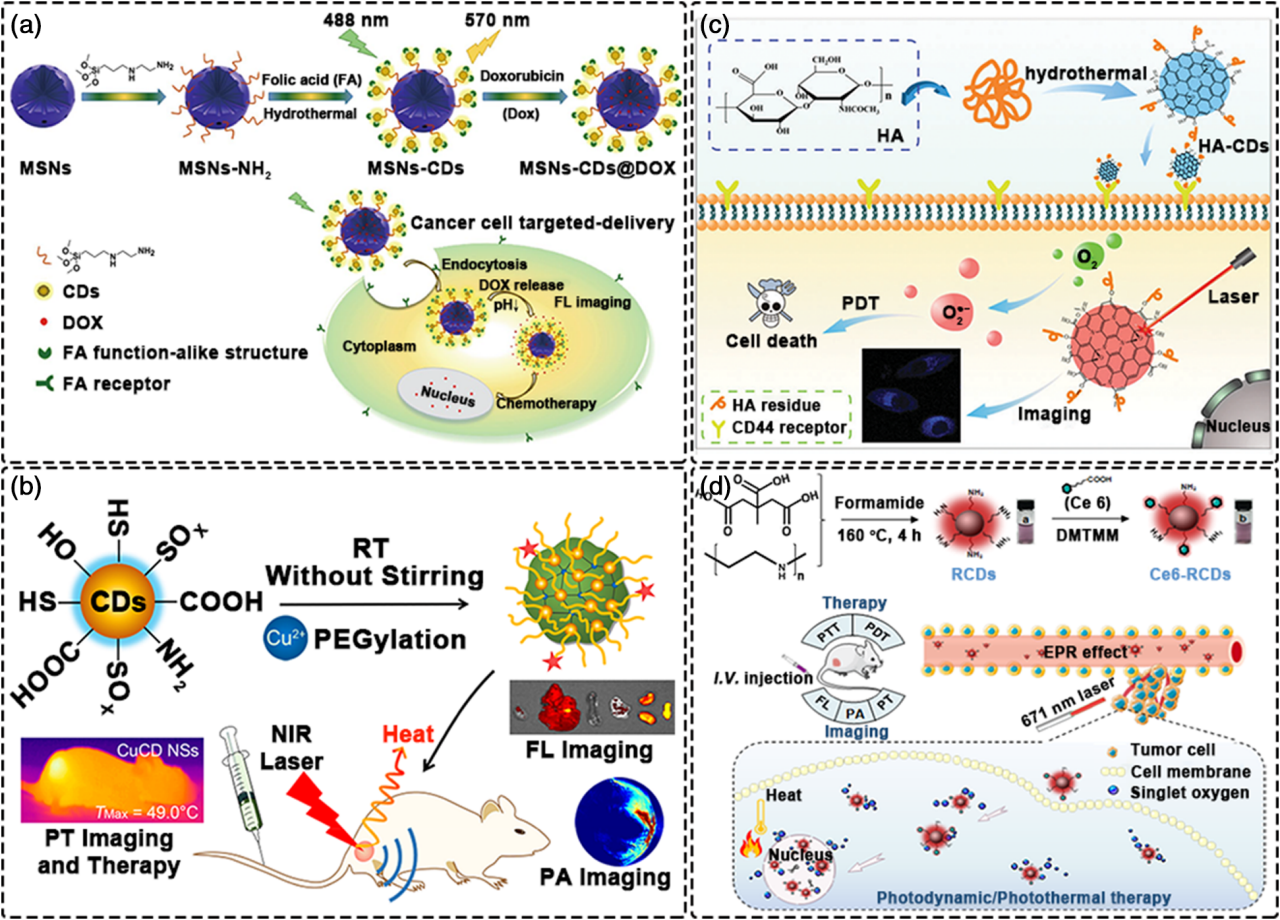
Synergistic therapy of CDs. a) Imaging‐guided drug delivery. Reproduced under the terms of the CC‐BY 4.0 license.^[^ ^276^ ^]^ Copyright 2019, The Authors, published by Springer. b) Multi‐modal imaging‐guided PTT therapy. Reproduced with permission.^[^ ^277^ ^]^ Copyright 2018, American Chemical Society. c) Imaging, drug delivery and PDT for synergistic therapy. Reproduced with permission.^[^ ^278^ ^]^ Copyright 2018, Royal Society of Chemistry. d) PTT/PDT integrated platform. Reproduced with permission.^[^ ^279^ ^]^ Copyright 2019, American Chemical Society.

Imaging‐guided treatment methods can overcome traditional issues in cancer therapies insufficient drug concentration, high systemic toxicity, severe side effects, and lack of monitoring. As a non‐invasive strategy, advanced image analysis technologies can be used for the early diagnosis of cancer and provide a reliable basis for personalized treatment.^[^ ^272^, ^273^, ^274^ ^]^ Imaging analysis enables direct observation of the behavior of therapeutic nanoparticles in the metabolic pathways and control of the response to external stimuli.^[^ ^275^ ^]^ For instance, the CDs modified with mesoporous silica exhibit strong yellow emission, thereby offering the potential for fluorescence imaging‐guided treatment and the ability to deliver drugs as fluorescence‐guided nanocarriers for targeted delivery (**Figure** **10**a).^[^ ^276^ ^]^ By combining Cu nanoparticles and sulfur‐doped CDs, Cu/CDs‐nanosheet in a one‐pot method, then coating the obtained Cu/CDs NS with thiol‐polyethylene glycol and fluorescent molecules, selective targeting of tumor tissues was achieved along with multi‐modal (photoacoustic, photothermal, and fluorescence) imaging‐guided PTT therapy (Figure 10b).^[^ ^277^ ^]^ In addition, Cu/CDs NS simultaneously exhibit laser‐triggered cytosolic delivery, lysosomal escape and nuclear targeting properties, greatly improving their therapeutic effects. The CDs prepared using hyaluronic acid (HA) can target cancer cells and selectively image cancer cells overexpressing CD44, efficiently generating O_2_^•−^ under 650 nm laser irradiation to inhibit cell growth (Figure 10c).^[^ ^278^ ^]^

In addition to imaging‐induced drug delivery, PTT and PDT, combination therapies using both PDT and PTT have emerged as a key direction of phototherapy in recent years. Compared with the traditional PTT/PDT integrated platforms that requires two different light sources, CDs‐based platforms offer multi‐modal treatment using only a single light source, resulting in the simultaneous generation of ROS and heat, thereby killing cancer cells more effectively. Loading a small amount of photosensitizer on the PTT agent can overcome the need for high‐power laser irradiation for effective PTT. As proof‐of‐concept, a small amount of photosensitizer Ce6 was anchored on CDs rich in amino groups. The CDs have excellent PTT characteristics (PCE = 46%) under 671 nm laser. At the same time, the 671 nm laser irradiation also activated the PDT of Ce6 (Figure 10d).^[^ ^279^ ^]^

### 5.6 Antiviral

The emergence of new viruses and their potential threat to humans is a global problem. The recent outbreaks of severe acute respiratory syndrome coronavirus 2 (SARS‐CoV‐2) and coronavirus disease 2019 (COVID‐19) serve as excellent examples of the need to develop effective antiviral therapies.^[^ ^280^, ^281^, ^282^ ^]^ Efficient antiviral strategies to be developed, rather than just depending on the traditional vaccination approach. Generally, viruses have sizes on nanoscale (typically 20–200 nm), which offers clues for constructing carbon‐based nanomaterials (e.g., CDs) on the same scale to impart antiviral effect.^[^ ^283^, ^284^, ^285^ ^]^ Though studies on the antiviral activities of CDs are in their embryonic stage, they will likely have an important future role in antiviral applications.

Virus infection involves attachment, cell penetration, replication and budding. Accordingly, well‐designed CDs should aim to inhibit viruses by blocking or suppressing infection processes at different stages.^[^ ^286^ ^]^ As the first step of inhibiting infection, well‐designed CDs can either directly interact with viruses, thus impeding virus‐cell interactions^[^ ^287^ ^]^ or inhibit the binding of virus to receptors,^[^ ^288^ ^]^ which may constitute a broad‐spectrum strategy to restrain viral infections. Once a virus approaches and attaches to the host cell, suppressing the penetration and entry to cells by employing functionalized CDs will be the best approach.^[^ ^289^, ^290^ ^]^ Though the mechanism of this action is still not completely understood, results and mechanistic studies suggest that CDs may act to suppress protein S‐receptor interactions within the host cell membrane. In the case of virus entry into cells, it is priority to destroy its replication and budding.^[^ ^291^ ^]^ For example, a series of curcumin‐derived CDs (Cur‐CDs) were synthesized through one‐step dry heating at different temperatures by Huang and co‐workers.^[^ ^292^ ^]^ The one‐step heating of curcumin at 180 °C retained many polymeric curcumin moieties on the surfaces of the Cur‐CDs, resulting in superior antiviral characteristics. The antiviral effect mainly involved inhibition of the translation of EV71‐ and EV71‐induced eIF4G cleavage and decreasing the expression of phosphorylated p38 kinase (almost completely). Taken together, these results encourage the development of CDs as agents for the treatment of virus infections.

## 6 Conclusions and Outlook

CDs are fascinating group of nanomaterials that have attracted increasing interest from the scientific community in the past decade. Due to their high stability, low toxicity and tunable optical properties, CDs are becoming very popular in biological and biomedical applications. Meanwhile, advances in the synthesis of CDs with novel structures creates functions. A deeper understanding of structure‐function relationships in CDs is needed in order to maximize their application value. In this review, we showcased some of the latest developments in the synthesis of CDs for bioimaging, biosensing, and as agents for antibacterial, anticancer and antiviral therapies.

The low toxicity of CDs allows their use in bio‐related applications. The fluorescence characteristics of CDs endow them with excellent imaging characteristics. In particular, CDs that can be excited by red or NIR light (i.e., wavelengths from 600 to 1000 nm) have assumed particular prominence in the field dues to their low damage to tissues during photoexcitation. The easy surface modification of CDs with analyte‐specific targeting sites provides a robust framework for optical sensor development (i.e., changes in the fluorescence intensity of CDs following interaction with an analyte). Meanwhile, the abundant functional groups on the surface allow CDs to deliver drug molecules to target cells and tissues for chemotherapy. Due to their different surface charge and the ability to generate ROS, CDs can also be used as specific or broad‐spectrum fungicides. The strong light absorption capacity of CDs enables photothermal and photodynamic therapies (and PTT/PDT combination therapies) to selectively destroy tumors or microorganisms. Although the bioapplication of CDs‐based materials has made great progress in the past decade, there are still some issues that need to be urgently addressed: 1) Although the methods for the preparation of CDs are diverse, the production of CDs at kilogram scales – as would be needed to commercial or clinical applications ‐ is difficult. Whilst microwave, hydrothermal, magnetic heating and other methods have used to achieve large‐scale production, production at a 1 kg scale or larger is technically challenging (often owing to the post‐synthetic purification steps needs to isolate the CDs from other synthesis byproducts). In addition, although supported template approaches can be used to synthesize CDs of desired sizes and properties, the etching of template using a strong acid or alkali to remove the sacrificial template and release the nanosized adds extra procedures and by‐products (thus extra costs). Methods for the synthesis of CDs in large quantities with specific properties and minimal waste or post synthetic processing steps are required; 2) In order to minimize light scattering and tissue damage, whilst also achieving deeper light penetration into tissues, CDs with deep red to NIR light excitation are needed for imaging, imaging‐guided therapy and phototherapies. To date, bioimaging with CDs has mainly focused on fluorescence imaging, which is of limited the value in biological tissues. Multimodal imaging, which use different imaging modes, such as combination of computed tomography, magnetic resonance imaging and fluorescence imaging, is therefore preferable. More work is needed to design and fabricate two‐/multiphoton or deep red/NIR excitation/emission CDs especially with multi‐modal imaging properties for biomedical applications; 3) Due to the fact that is difficult for CDs to enter the nucleus of cells, most of the cytotoxicity studies about CDs have been focused on the cytoplasmic level. Therefore, there is currently little information about whether CDs can damage the nucleus. The risk of CDs‐induced genotoxicity needs to be fully investigated. For in vivo toxicity, the commonly used animal models (such as mice, zebrafish, etc.) may not be able to mimic accurately the complexity of the human body environment. It is necessary to conduct toxicity studies on non‐human primates to explore their potential clinical translational value; 4) The bio‐environment is a very complex system, which contains not only the target analyte but also many similar substances. Biosensing using CDs can therefore be negatively impacted by strong interference (chemical and sometimes optical). Although surface modification and functionalization of CDs can improve the targeting selectivity, the functional groups on the surface of CDs may also interact with some substances in the bio‐environment, leading to false signals. Hence, the type of functional groups on the surface of CDs need to be chosen carefully for specific applications (after considering all endogenous molecules/tissues the CDs might interact with); 5) In the future, the targeted delivery of CDs‐loaded therapeutic drugs will gain increasing importance. Molecular recognition of multiple cell receptors overexpressed in the target region will be a key consideration. In addition, the sustained release and controlled release of specific drugs in the lesion demands further study. Currently, the understanding of the in vivo behavior of CDs drugs is very limited, with clinical trials desperately needed; 6) The low accumulation of nano‐reagents and weak light‐to‐heat conversion reduce the PTT efficiency of CDs. As a local treatment method, the effect of PTT is mostly limited to the irradiated area. In non‐irradiated areas, tumor cells can survive in PTT, which will inevitably lead to recurrence and metastasis. In order to solve these problems, CDs that make full use of NIR‐II light should be developed to offset the energy dissipation to deep tissues; 7) The phototoxicity of currently used CDs and CDs@PS systems is dependent on the O_2_ concentration, which makes effective PDT therapy difficult in the hypoxic tumor environment. Meanwhile, in the aerobic lesions, PDT continuously consumes O_2_, which intensifies the tumor's hypoxia and aggravates the tumor's resistance to PDT. Therefore, developing photosensitizer systems that do not rely on O_2_ or which can alleviate oxygen consumption is essential for more effective cancer treatment; 8) For the nanomedicine applications of CDs, combining the functions of treatment and diagnosis is a priority. Current collaborative treatments such as imaging‐guided therapy and PDT/PTT coordination serve as an effective example of what is possible using rationally‐designed nanomaterials. To date, there are few treatment reports about real‐time monitoring using CDs. This would represent an efficient and time‐saving method for evaluating the health status of patients, and deserves further attention in the future; 9) Research into CDs‐based antivirals is still in its infancy. At present, it is a challenge to target and inhibiting specific viruses using CDs. In addition, it is necessary to accommodate the mutation of viruses and tailor CDs to combat each new virus strain as it emerges; and 10) Recently, the scientific community has witnessed significant breakthroughs in single‐atom nanotherapy for versatile bioapplications. However, the bioapplications research of CDs‐based single‐atom nanomedicine has just started. How to improve the loading efficiency of individual metal atoms, enhance the selectivity of complexes, and clarify the exact mechanism for bioapplications is crucial.

Despite the above‐mentioned challenges, the advantages of CDs over other nanomaterials (e.g., noble metal nanoparticles) in many areas of biology and medicine are becoming very obvious. This motivates researchers in many fields, including nanomedicine, synthetic chemistry, materials science and other fields. Through better understanding of the properties of CDs, and new syntheses which will allow CDs with specific/precisely tailored properties to be produced at scale, it is expected that CDs will take a frontline role in the detection and treatment of a range of threats in the near‐future.

## Conflict of Interest

The authors declare no conflict of interest.
